# Sample size determination for hypothesis testing on the intraclass correlation coefficient in a two‐way analysis of variance model

**DOI:** 10.1111/bmsp.70016

**Published:** 2025-11-14

**Authors:** Dipro Mondal, Alberto Cassese, Math J. J. M. Candel, Sophie Vanbelle

**Affiliations:** ^1^ Department of Methodology and Statistics Care and Public Health Research Institute (CAPHRI), Maastricht University Maastricht The Netherlands; ^2^ Department of Statistics Computer Science, Applications “G. Parenti”, The University of Florence Florence Italy

**Keywords:** Interrater reliability, Measurement errors, Observer variation, Power, Reproducibility of results, Sample size calculation

## Abstract

Reliability evaluation is critical in fields such as psychology and medicine to ensure accurate diagnosis and effective treatment management. When participants are evaluated by the same raters, a two‐way ANOVA model is suitable to model the data, with the intraclass correlation coefficient (ICC) serving as the reliability metric. In these domains, the ICC for agreement (ICCa) is commonly used, as the values of the measurements themselves are of interest. Designing such reliability studies requires determining the sample size of participants and raters for the ICCa. Although procedures for sample size determination exist based on the expected width of the confidence interval for the ICCa, there is limited work on hypothesis testing. This paper addresses this gap by proposing procedures to ensure sufficient power to statistically test whether the ICCa exceeds a predetermined value, utilizing confidence intervals for the ICCa. We compared the available confidence interval methods for the ICCa and proposed sample size procedures using the lower confidence limit of the best performing methods. These procedures were evaluated considering the empirical power of the hypothesis test under various parameter configurations. Furthermore, these procedures are implemented in an interactive R shiny app, freely available to researchers for determining sample sizes.

## 1 INTRODUCTION

The integration of measurement instruments and procedures in psychology and medicine into daily practice and research underscores the importance of ensuring that these instruments are both reliable (i.e. the instrument/rater is capable of distinguishing among participants in a population) and valid (i.e. the outcome measurements reflect the underlying true values) (Souza et al., 2017). The reliability of measurement instruments is typically evaluated in intra‐rater or inter‐rater reliability studies, which quantify the reliability using the intraclass correlation coefficient (ICC) for quantitative outcomes. The ICC can take different forms depending on the study design. This paper focuses on inter‐rater reliability studies, where each participant is measured once by every rater involved. A two‐way analysis of variance (ANOVA) model is suitable to model the measurements in such studies. Under this model, two ICC coefficients can be computed: the ICC for agreement and the ICC for consistency. The choice between these coefficients depends on whether systematic differences between raters are relevant. The ICC for consistency ignores systematic differences among raters, focusing solely on expressing to what extent raters have the same rank order of participants. In contrast, the ICC for agreement considers both the rank ordering of participants and systematic differences between raters, as it is based on absolute measurements. In disciplines such as psychology and medicine, the interest often extends beyond the rank ordering of participants. Furthermore, since the values provided by measurement instruments are used to guide decisions or develop treatment plans, the ICC for agreement is commonly preferred and is therefore the focus of this paper.

During the planning stage of a reliability study, determining an adequate sample size for the number of participants and raters is essential to draw reliable conclusions. A review of the literature revealed three different sample size determination procedures for calculating the number of participants, raters or their combination (Dobbin & Ionan, 2015; Doros & Lew, 2010; Saito et al., 2006). These procedures aim to ensure a certain precision around the ICC estimate (see Mondal et al. (2025) for a review of these methods). However, rather than focusing on achieving a specific level of precision around the ICC estimate, a researcher may instead be interested in statistically testing whether the ICC exceeds an acceptable value. In this case, sample sizes must be determined to achieve a predefined power for the hypothesis test, given a specified type‐I error rate.

The sample size determination procedure for hypothesis testing concerning the ICC for agreement relies on the lower limit of a one‐sided confidence interval (Mondal et al., 2024; Zou, 2012). A literature review identifies seven different classes of confidence interval methods: the moment approximation method (Fleiss & Shrout, 1978; Zou & McDermott, 1999), the modified profile likelihood method (Xiao et al., 2010), the delta method (Almehrizi & Emam, 2021; Saito et al., 2006), the modified large sample method (Arteaga et al., 1982; Cappelleri & Ting, 2003), the generalized confidence interval method (Weerahandi, 1993), the variance partitioning method (Demetrashvili et al., 2016) and a confidence interval method based on asymptotic normality (Bourredjem et al., 2024). A recent study (Bourredjem et al., 2024) proposed a sample size determination procedure for hypothesis testing that utilizes the asymptotic normality of the ICC for agreement. However, the statistical properties of confidence intervals based on normality are known to be poor, particularly for small sample sizes (Bourredjem et al., 2024; Mondal et al., 2024). In addition, the study does not consider all the available confidence interval methods. To address these limitations, we propose and develop sample size procedures that incorporate all available confidence interval methods.

Our contribution is fivefold. First, we compare the empirical type‐I error rates of the available confidence interval methods via a Monte Carlo simulation study, specifically focusing on one‐sided confidence intervals for the ICC for agreement. Second, we develop sample size determination procedures based on the confidence interval methods that demonstrated acceptable type‐I error rates to achieve a predefined power for hypothesis testing of the ICC for agreement. Power functions leading to the calculation of sample sizes will also be developed. Third, we evaluate the proposed sample size determination procedures by assessing the empirical power at the calculated sample sizes. Fourth, we provide guidelines to select the best method and its corresponding sample size procedure. Fifth, we develop an R‐shiny app with a user‐friendly, menu‐driven interface to facilitate the calculation of sample sizes using the procedures outlined in this paper.

The paper is organized as follows. Section 2 introduces the ICC for agreement within the context of a reliability study, where each participant is evaluated once by every rater, with the data modelled using a two‐way ANOVA model. This section also presents the hypothesis test framework and describes the various methods used for constructing confidence intervals within this context. Section 3 outlines a Monte Carlo simulation study designed to compare the empirical type‐I error rates of the different confidence interval methods. Section 4 proposes a procedure for determining sample sizes for hypothesis testing related to the ICC for agreement, including the derivation of power functions for the confidence interval methods. Section 5 calculates sample sizes using the proposed procedure and evaluates them in terms of empirical power through a Monte Carlo simulation study. Section 6 offers guidance on parameter selection for sample size determination. Section 7 demonstrates the application of the sample size determination procedures within the context of a specific reliability study. Finally, Section 8 discusses the various methods and procedures described throughout the paper.

## 2 DEFINITIONS

Consider a study in which n participants are rated once by k raters using the same measurement instrument. The rating of a participant i(i=1,2,..,n) by a rater j(i=1,2,..,k) can be described by a two‐way ANOVA model,

(1) $$Y_{ij}=\mu +s_{i}+r_{j}+e_{ij},$$

where μ is the grand mean, $s_{i}$ is the random effect ($s_{i}∼𝒩(0,\sigma_{s}^{2})$) of participant i, $r_{j}$ is the random effect ($r_{j}∼𝒩(0,\sigma_{r}^{2})$) of rater j and $e_{ij}$ denotes the error ($e_{ij}∼𝒩(0,\sigma_{e}^{2})$). Since, in the study, the raters rate a participant only once, an interaction effect cannot be estimated and is therefore excluded from the model. Under the ANOVA model in Equation (1), one can obtain the ICC for agreement from the variance components as (McGraw & Wong, 1996),

(2) $$\rho =\frac{\sigma_{s}^{2}}{\sigma_{s}^{2}+\sigma_{r}^{2}+\sigma_{e}^{2}},\;\;0\leq \rho \leq 1\;.$$

An important quantity of interest is the rater‐to‐error variance ratio, denoted by $R=\frac{\sigma_{r}^{2}}{\sigma_{e}^{2}}$.

The variance component estimates are derived from the mean squares of the ANOVA model (Equation 1). Specifically, the mean square between participants, MSS $\left(=\frac{k}{n-1}\sum_{i=1}^{n}{\left({\bar{y}}_{i.}-{\bar{y}}_{..}\right)}^{2}\right)$, the mean square between raters, MSR $\left(=\frac{n}{k-1}\sum_{j=1}^{k}{\left({\bar{y}}_{.j}-\bar{y}..\right)}^{2}\right)$ and the mean square of errors, MSE $\left(=\frac{1}{\left(n-1)(k-1\right)}\sum_{i=1}^{n}\sum_{j=1}^{k}{\left(y_{ij}-{\bar{y}}_{i.}-{\bar{y}}_{.j}+{\bar{y}}_{..}\right)}^{2}\right)$, where ${\bar{y}}_{i.}=\frac{1}{k}\sum_{j=1}^{k}y_{ij}$ (the estimated mean for participant i), ${\bar{y}}_{.j}=\frac{1}{n}\sum_{i=1}^{n}y_{ij}$ (the estimated mean for rater j) and ${\bar{y}}_{..}=\frac{1}{nk}\sum_{i=1}^{n}\sum_{j=1}^{k}y_{ij}$ (the estimated overall mean). Table 1 displays these different mean squares and their expected values.

**TABLE 1:** Variance decomposition for the two‐way ANOVA model (Equation 1).

Sources of Variation	Degrees of Freedom	Mean Square	Expected Mean Square
Between participants	n−1	MSS	$k\sigma_{s}^{2}+\sigma_{e}^{2}$
Between raters	k−1	MSR	$n\sigma_{r}^{2}+\sigma_{e}^{2}$
Errors	(n−1)(k−1)	MSE	$\sigma_{e}^{2}$

The ICC for agreement, as defined in Equation (2), can then be estimated by applying the method of moments to estimate the variance components. The estimator is (Bartko, 1966; McGraw & Wong, 1996)

(3) $$\hat{\rho }=\frac{MSS-MSE}{MSS+(k-1)MSE+\frac{k}{n}(MSR-MSE)}.$$

### 2.1 Hypothesis testing concerning the ICC for agreement

This paper focuses on determining the sample size required for a reliability study in the context of hypothesis testing concerning ρ, ensuring that certain conditions are satisfied. Specifically, consider the scenario where one is interested in testing a hypothesis about ρ, with the null hypothesis stating that ρ is less than or equal to the value under the null hypothesis, $\rho_{0}$, that is, $\rho \leq \rho_{0}$, and the alternative hypothesis stating that ρ is greater than $\rho_{0}$, that is, $\rho >\rho_{0}$. Let $\rho_{A}$ represent the value of ρ under the alternative hypothesis. The power of this hypothesis test, 𝒫(n), is the probability of rejecting the null hypothesis when $\rho =\rho_{A}$. The required sample size, n, is then determined to ensure that the power of the hypothesis test is as close as possible to a predefined threshold, 1−β, while not falling below it. For a given number of raters, k, the rater‐to‐error variance ratio, R, type‐I error rate, α and values $\rho_{0}$ and $\rho_{A}$, the minimum number of participants, n, to achieve a power larger than 1−β, is obtained by satisfying the following criterion (Mondal et al., 2024; Zou, 2012),

(4) $$P[L>\rho_{0}|\rho =\rho_{A}]\geq 1-\beta ,$$

where L is the lower limit of the one‐sided confidence interval for ρ. The following section gives an overview of the different confidence interval methods for ρ.

### 2.2 Confidence interval methods for the ICC for agreement

Seven classes of confidence interval methods are available in the literature to construct a confidence interval for ρ (see Mondal et al. (2025) for a review of six of these classes), with only a few of them showing good statistical properties. Comparison studies (Cappelleri & Ting, 2003; Ionan et al., 2014; Rousson et al., 2003; Tian & Cappelleri, 2004; Xiao et al., 2010; Zou & McDermott, 1999) investigating the statistical properties of two‐sided confidence intervals for a subset of available methods conclude that the modified large sample confidence interval method by Gui et al. (1995), adapted for ρ by Cappelleri and Ting (denoted as $MLS_{G}$) (Cappelleri & Ting, 2003), and the generalized confidence interval method (denoted by GCI) (Dobbin & Ionan, 2015; Weerahandi, 1993) provide coverage probabilities closest to the nominal coverage under most scenarios. These scenarios include various combinations of the number of participants (n) and raters (k), the ratio of rater to error variances (R) and the ICC for agreement (ρ). Expanding these comparisons, Mondal et al. (2025) further found that the variance partitioning confidence interval proposed by Demetrashvili et al. (2016), especially the one using an F‐distribution ($VP_{F}$), provided coverage probabilities as good as $MLS_{G}$ and GCI when the rater‐to‐error variance ratio, R is less than or equal to .1. However, since these conclusions are drawn for two‐sided confidence intervals, they may not directly apply to one‐sided confidence intervals, as also confirmed by our results. This is because confidence intervals for ρ are generally not symmetric (Liljequist et al., 2019), and different statistical criteria are used to evaluate one‐sided intervals. For example, while the performance of two‐sided confidence intervals deteriorates as ρ decreases (see Mondal et al., 2025; Section 4), we observe the opposite trend for one‐sided confidence intervals. Therefore, in this paper, we compare the performance of one‐sided intervals for a selection of the better‐performing methods. Specifically, we compare the confidence interval based on the delta method and matrix formulation (Almehrizi & Emam, 2021) (denoted as $W_{mat}$), the modified large‐sample confidence interval based on a transformation of ρ (Arteaga et al., 1982) (denoted as $MLS_{A}$), the modified large‐sample confidence interval based on variance component ratios (Cappelleri & Ting, 2003) (denoted as $MLS_{G}$), the generalized confidence interval (Weerahandi, 1993) (denoted as GCI), the variance partitioning confidence interval (Demetrashvili et al., 2016) using an *F*‐distribution (denoted as $VP_{F}$) and a beta‐distribution (denoted as $VP_{B}$). We further include in the comparison the confidence interval method based on asymptotic normality of the variance estimators recently proposed by Bourredjem et al. (Bourredjem et al., 2024) (denoted as ASN), which has not been included in any of the previous studies. To utilize these confidence intervals for sample size determination in a one‐sided hypothesis test, commonly used in reliability testing, we focus on the lower limit of the one‐sided confidence interval. In this case, the upper limit is set to 1, the maximum possible value for ρ. The following subsections describe how the lower limit is determined using these methods.

#### 2.2.1 Confidence interval based on the delta method and matrix formulation ($W_{mat}$)

Almehrizi and Emam (Almehrizi & Emam, 2021) utilized the delta method to obtain the (1−α)×100% one‐sided lower confidence limit for ρ as follows,

(5) $$L=\hat{\rho }-T_{n+1}(1-\alpha )\sqrt{\hat{V}(\hat{\rho })},$$

where $T_{n+1}(1-\alpha )$ is the (1−α)×100 percentile of the t‐distribution with n+1 degrees of freedom. In Equation (5), $\hat{V}(\hat{\rho })$ is obtained as,

(6) $$\begin{array}{cc} \hat{V}(\hat{\rho })= & \frac{2{\hat{\rho }}^{2}\left[{\left(1+\frac{nk-n-k}{n}\hat{\rho }\right)}^{2}trS^{2}+{\left(1-\frac{\hat{\rho }}{n}\right)}^{2}{\left(1^{\prime }S1\right)}^{2}-2\left(1+\frac{nk-n-k}{n}\hat{\rho }\right)\left(1-\frac{\hat{\rho }}{n}\right)1^{\prime }S^{2}1\right]}{(n+1){\left[1^{\prime }S1-trS\right]}^{2}}\\ & +\frac{8k{\hat{\rho }}^{4}y_{d}^{\prime }y_{d}}{{\left[1^{\prime }S1-trS\right]}^{2}}, \end{array}$$

where 1 is a row vector of 1's (k×1), S is the estimated sample variance‐covariance matrix of the k measurements (k×k), and $y_{d}={\left({\bar{y}}_{.1}-{\bar{y}}_{..},\text{…},{\bar{y}}_{.k}-{\bar{y}}_{..}\right)}^{\prime }$ is a (k×1) vector of deviations of the average measurements for each rater j (${\bar{y}}_{.j}$) from the grand average (${\bar{y}}_{..}$). Note that, $1^{\prime }S1=kMSS=\frac{k^{2}}{n-1}\sum_{i=1}^{n}{\left({\bar{y}}_{i.}-{\bar{y}}_{..}\right)}^{2}$ is the sample variance of the total measurements across all participants, $trS=MSS+(k-1)MSE=\sum_{j=1}^{k}\left(\frac{1}{n-1}\sum_{i=1}^{n}{\left(y_{ij}-{\bar{y}}_{.j}\right)}^{2}\right)$ is the sum of within rater sample variances, and, $y_{d}^{\prime }y_{d}=\frac{k-1}{n}MSR=\sum_{j=1}^{k}{\left({\bar{y}}_{.j}-{\bar{y}}_{..}\right)}^{2}$ is the sum of squared deviations of average measurements of each rater j from the grand average.

#### 2.2.2 Modified large sample confidence interval based on a transformation of ρ ($MLS_{A}$)

The modified large sample confidence interval is a framework for constructing a confidence interval for a linear combination of the variance components (Burdick et al., 2005; Graybill & Wang, 1980; Gui et al., 1995). Arteaga et al. (1982) utilized this method to obtain the (1−α)×100% one‐sided lower confidence limit for the transformation, $\frac{k\rho }{n(1-\rho )}$ as (Arteaga et al., 1982),

(7) $$L_{A}^{*}=\frac{-1+F_{n-1,\infty }^{-1}(1-\alpha )F_{1}+(1-F_{n-1,\infty }^{-1}(1-\alpha )F_{4})F_{4}F_{1}^{-1}}{n-1+F_{n-1,\infty }^{-1}(1-\alpha )F_{3}F_{2}},$$

where $F_{1}=\frac{MSS}{MSE}$, $F_{2}=\frac{MSR}{MSE}$, $F_{3}=F_{n-1,k-1}\left(1-\alpha \right)$, $F_{4}=F_{n-1,(n-1)(k-1)}\left(1-\alpha \right)$. $F_{df_{1},{df}_{2}}(1-\alpha )$ denotes the (1−α)×100 percentile of an F‐distribution with ${df}_{1}$ and ${df}_{2}$ degrees of freedom in the numerator and denominator, respectively. Then, the lower limit for the confidence interval for ρ is,

$$L=\frac{n\;\max(0,L_{A}^{*})}{k+n\;\max(0,L_{A}^{*})}.$$

#### 2.2.3 Modified large sample confidence interval based on the ratio of variance components ($MLS_{G}$)

The general framework for constructing a confidence interval for the ratio of variance components, as proposed by Gui et al. (1995), was adapted by Cappelleri and Ting (2003) for ρ, which represents a ratio of a linear combination of variance components. Defining

(8) $$\begin{array}{cc} & H_{i}=1/F_{d_{i},\infty }(\alpha )-1\;\forall i\in 1,2,3,\\ & G_{i}=1-1/F_{d_{i},\infty }(1-\alpha )\;\forall i\in 1,2,3,\;\text{and}\\ & G_{1j}=[{\left(1-F_{d_{1},d_{j}}(1-\alpha )\right)}^{2}-{\left(G_{1}F_{d_{1},d_{j}}(1-\alpha )\right)}^{2}-H_{j}^{2}]/F_{d_{1},d_{j}}\forall j\in 2,3, \end{array}$$

where $d_{1}$, $d_{2}$ and $d_{3}$ are respectively, n−1, k−1 and (n−1)(k−1). Then, the (1−α)×100% one‐sided lower confidence limit for ρ is given by,

(9) $$L=\frac{-B_{L}-\sqrt{Q_{L}}}{2A_{L}},$$

where $Q_{L}=max(0,B_{L}^{2}-2A_{L}C_{L})$, in which

$$\begin{array}{cc} & A_{L}=[{\left(1-G_{1}\right)}^{2}MSS^{2}+(1-H_{2}^{2}){\left(k/n\right)}^{2}MSR^{2}+{\left(1-H_{3}\right)}^{2}{\left(k-1-k/n\right)}^{2}MSE^{2}+\\ & (2+G_{12})(k/n)MSS\times MSR+(2+G_{13})(k-1-k/n)MSS\times MSE+\\ & 2(k/n)(k-1-k/n)MSR\times MSE],\\ & B_{L}=[-2{\left(1-G_{1}\right)}^{2}MSS^{2}+2{\left(1-H_{3}\right)}^{2}{\left(k-1-k/n\right)}^{2}MSE^{2}-\\ & (2+G_{12})(k/n)MSS\times MSR-(2+G_{13})(k-2-k/n)MSS\times MSE+\\ & 2(k/n)MSR\times MSE],\;\text{and},\\ & C_{L}=(1-G_{1}^{2})MSS^{2}+(1-H_{3}^{2})MSE^{2}-(2+G_{13})MSS\times MSE. \end{array}$$

#### 2.2.4 Generalized confidence interval (GCI)

A general method for constructing confidence intervals was proposed by Weerahandi (Weerahandi, 1993), based on the concept of a generalized pivotal quantity (Tsui & Weerahandi, 1989; Weerahandi, 1993). The generalized pivotal quantity for ρ is given by Tian and Cappelleri (2004),

(10) $$g(t)=\frac{max\left[0,\frac{(n-1)MSS}{k\chi_{n-1}^{2}}-\frac{(n-1)(k-1)MSE}{k\chi_{\left(n-1)(k-1\right)}^{2}}\right]}{\frac{(k-1)MSR}{n\chi_{k-1}^{2}}+\frac{(n-1)MSS}{k\chi_{n-1}^{2}}+\frac{(nk-n-k+1)(nk-n-k)MSE}{nk\chi_{\left(n-1)(k-1\right)}^{2}}},$$

where t denotes a vector of mean squares. The (1−α)×100% one‐sided lower confidence limit for ρ is given by the quantile function Ĝ as (Dobbin & Ionan, 2015; Ionan et al., 2014; Tian & Cappelleri, 2004)

$$L=Ĝ\left(\alpha \right),$$

which is the empirical quantile of the pivotal quantity in Equation (10) that can be obtained through sampling methods. The process involves sampling from $\chi^{2}$ distributions and plugging in the values so obtained in Equation (10) (Dobbin & Ionan, 2015; Ionan et al., 2014; Tian & Cappelleri, 2004). This Monte Carlo step should be repeated several times, since the accuracy of the method grows as the number of Monte Carlo samples increases. It is recommended to draw at least 100,000 samples (Burdick et al., 2005; Dobbin & Ionan, 2015) such that the difference between Ĝ and G becomes negligible.

#### 2.2.5 Variance partitioning confidence interval using an F‐distribution ($VP_{F}$)

The variance partitioning method for ρ was proposed by Demetrashvili et al. (Demetrashvili et al., 2016). This method involves partitioning the three variance components (see Table 1) into two partitions, depending on whether the variance components involve the rater process. When this is the case, the two partitions are $\sigma_{s}^{2}$ and $\sigma_{E}^{2}=\sigma_{r}^{2}+\sigma_{e}^{2}$. Denoting the total variance as $\sigma_{T}^{2}=\sigma_{s}^{2}+\sigma_{E}^{2}$, the estimated variance of the estimator of ρ is given by (Demetrashvili et al., 2016) as,

(11) $$\begin{array}{c} \hat{V}(\hat{\rho })\approx \frac{{\hat{\sigma }}_{E}^{4}}{{\left({\hat{\sigma }}_{T}^{2}\right)}^{4}}\hat{V}({\hat{\sigma }}_{s}^{2})+\frac{{\hat{\sigma }}_{s}^{4}}{{\left({\hat{\sigma }}_{T}^{2}\right)}^{4}}\hat{V}({\hat{\sigma }}_{E}^{2})-\frac{2{\hat{\sigma }}_{s}^{2}{\hat{\sigma }}_{E}^{2}}{{\left({\hat{\sigma }}_{T}^{2}\right)}^{4}}\hat{Cov}({\hat{\sigma }}_{s}^{2},{\hat{\sigma }}_{E}^{2}), \end{array}$$

where $\hat{V}({\hat{\sigma }}_{E}^{2})=\hat{V}({\hat{\sigma }}_{r}^{2})+\hat{V}({\hat{\sigma }}_{e}^{2})+2\hat{Cov}({\hat{\sigma }}_{r}^{2},{\hat{\sigma }}_{e}^{2})$ and the generic quantity $\hat{Cov}({\hat{\sigma }}_{i}^{2},{\hat{\sigma }}_{j}^{2})$ is the estimated covariance of the estimators ${\hat{\sigma }}_{i}^{2}$ and ${\hat{\sigma }}_{j}^{2}$.

In this method, it is assumed that the covariance between the variance estimators are 0 and that, $\frac{{\hat{\sigma }}_{s}^{2}}{{\hat{\sigma }}_{E}^{2}}$ is distributed as ${\left(\frac{1}{\hat{\rho }}-1\right)}^{-1}f_{{\hat{df}}_{1},{\hat{df}}_{2}}$ where $f_{{\hat{df}}_{1},{\hat{df}}_{2}}$ denotes the probability density of an F‐distribution with ${\hat{df}}_{1}$ degrees of freedom in the numerator and ${\hat{df}}_{2}$ degrees of freedom in the denominator. The degrees of freedom are ${\hat{df}}_{1}=\frac{2{\hat{\sigma }}_{s}^{4}}{\hat{V}({\hat{\sigma }}_{s}^{2})}$ and ${\hat{df}}_{2}=\frac{2{\hat{\sigma }}_{E}^{4}}{\hat{V}({\hat{\sigma }}_{E}^{2})}$, respectively (Demetrashvili et al., 2016). The (1−α)×100% one‐sided lower confidence limit for ρ is then given as,

(12) $$L=\frac{{\hat{\sigma }}_{s}^{2}F_{{\hat{df}}_{1},{\hat{df}}_{2}}(\alpha )}{{\hat{\sigma }}_{s}^{2}F_{{\hat{df}}_{1},{\hat{df}}_{2}}(\alpha )+{\hat{\sigma }}_{E}^{2}}.$$

where $F_{{\hat{df}}_{1},{\hat{df}}_{2}}(\alpha )$ denotes the α×100 percentile of an F‐distribution with ${\hat{df}}_{1}$ and ${\hat{df}}_{2}$ degrees of freedom in the numerator and denominator, respectively.

#### 2.2.6 Variance partitioning confidence interval using a beta‐distribution ($VP_{B}$)

This method utilizes the same variance partitioning method described for $VP_{F}$. In this method, however, the distribution of $\hat{\rho }$ is approximated by a beta‐distribution with shape parameters a>0 and b>0. The parameters are estimated as

(13) $$\begin{array}{cc} & â=\frac{\hat{\rho }[\hat{\rho }(1-\hat{\rho })-\hat{V}(\hat{\rho })]}{\hat{V}(\hat{\rho })},\\ & \hat{b}=\frac{\left(1-\hat{\rho })[\hat{\rho }(1-\hat{\rho })-\hat{V}(\hat{\rho })\right]}{\hat{V}(\hat{\rho })}, \end{array}$$

where $\hat{\rho }$ and $\hat{V}(\hat{\rho })$ are obtained from Equations 3 and 11, respectively. Modelling the distribution of $\hat{\rho }$ using a beta‐distribution with the parameters estimated as in Equation (13) may lead to some complications, and Demetrashvilli et al. (Demetrashvili et al., 2016) suggest corrections to avoid these problems. Specifically,

1 We expect the distribution of $\hat{\rho }$ to be unimodal, which is not the case for a beta‐distribution when both the shape parameters are less than 1 (a,b<1) leading to a U‐shaped distribution. In such a case, the beta‐distribution is transformed to a J‐shaped distribution by increasing the mass closer to 0 or 1, depending on whether $\hat{\rho }<.5$ or $\hat{\rho }>.5$. Specifically, two adjustments are made as mentioned below.
  - When $0<â,\hat{b}<1$ and $\hat{\rho }=\frac{â}{â+\hat{b}}<.5$, $\hat{b}$ is fixed at 1 and â is estimated by Equation (13).
  - When $0<â,\hat{b}<1$ and $\hat{\rho }=\frac{â}{â+\hat{b}}>.5$, â=1 is fixed at 1 and $\hat{b}$ is estimated by Equation (13).
2. Furthermore, Equation (13) may lead to negative estimates of a and b when the variance of $\hat{\rho }$ (Equation 11) is larger than $\hat{\rho }(1-\hat{\rho })$. Since a beta‐distribution is defined for positive parameters, two fixes are proposed in this case, depending on whether $\hat{\rho }$ is greater than or lesser than .5, following the same transformations mentioned earlier.
  - When $\hat{\rho }\leq .5$, $\hat{b}$ is fixed at 1 ($\hat{b}=1$) and â is obtained as $â=\hat{\rho }/(1-\hat{\rho })$.
  - When $\hat{\rho }>.5$, â is fixed at 1 (â=1) and $\hat{b}$ is obtained as $\hat{b}=\hat{\rho }/(1-\hat{\rho })$.

Then the (1−α)×100% lower confidence limit for ρ is given by

(14) $$L=B_{â,\hat{b}}(\alpha )$$

where $B_{â,\hat{b}}(\alpha )$ is the α×100 percentile of a beta‐distribution with parameters â and $\hat{b}$.

#### 2.2.7 Asymptotically normal confidence interval (ASN)

Bourredjem et al. (Bourredjem et al., 2024) proposed a confidence interval utilizing the asymptotic normality of the variance estimators. Then asymptotically $\hat{\rho }$ is also normally distributed (Bourredjem et al., 2024), and the (1−α)×100% lower confidence limit for ρ is given by,

(15) $$L=\hat{\rho }-z_{1-\alpha }\sqrt{2\frac{{\hat{\rho }}^{4}}{n}\left[{\left(\frac{1}{\hat{\rho }}-1\right)}^{2}+\frac{n}{k}\frac{{\hat{\sigma }}_{r}^{4}}{{\hat{\sigma }}_{s}^{4}}\right]},$$

where $z_{1-\alpha }$ is the (1−α)×100% percentile of a standard normal distribution.

## 3 COMPARISON OF CONFIDENCE INTERVAL METHODS

We conducted a Monte Carlo simulation study to compare the statistical properties of the methods mentioned above for one‐sided confidence intervals. Specifically, we compare the empirical type‐I error rates of the different methods to the theoretical one, under various scenarios. The following subsections describe the design of the Monte Carlo simulation study, discuss the results obtained in terms of type‐I error rates, and offer recommendations for the choice of the best performing confidence interval methods under different scenarios.

### 3.1 Design of the simulation study

The following steps describe the process for simulating a single dataset in a given scenario, which is defined by a combination of the ICC for agreement, ρ, the rater‐to‐error variance ratio, R, the type‐I error rate, α, the number of participants, n and raters, k. Following the ANOVA model in Equation (1), and without loss of generality, N=n×k errors are drawn from a normal distribution with mean 0 and variance $\sigma_{e}^{2}=1$. Then, k rater effects ($r_{j}$) are drawn for n participants from a normal distribution with mean 0 and variance $\sigma_{r}^{2}=R\times \sigma_{e}^{2}=R$. Finally, n participant effects ($s_{i}$) are drawn for each of the k raters from a normal distribution with mean 0 and variance $\sigma_{s}^{2}=\frac{\rho (R+1)}{1-\rho }$, since we set $\sigma_{e}^{2}=1$. For each scenario, $10^{5}$ datasets are generated.

In order to study the performance in terms of type‐I error rates, we consider $\rho_{0}=\rho$ and compute the empirical type‐I error rate as the proportion of times the lower limit of the confidence interval is greater than or equal to $\rho_{0}$. The empirical type‐I error rate of the confidence intervals is studied for various scenarios. Specifically, we choose $\rho_{0}=\rho \in \{.7,.8,.9\}$, since large ρ are usually of interest in a reliability study. Since empirical evidence from several reliability studies (Hernandez Engelhart et al., 2024; Bland & Altman, 1999; Cutti et al., 2010; Dobbin & Ionan, 2015; Fleiss & Shrout, 1978; Hart, 2019; Streiner & Norman, 1995; Ting et al., 2009; Carstensen et al., 2013) indicates that R≤1, we choose R∈{.01,.05,[.1−1],2,5} where the range from .1 to 1 is covered in steps of .1. For the number of participants and raters, we chose n∈{10,20,40,60,100,200} and k∈{2,3,4,5,8,10,15,20}, respectively, covering a broad range for the number of participants and raters. The nominal type‐I error rate, α is fixed at .05. Under the various scenarios described above, we examine how far the empirical type‐I error rates obtained using the confidence interval methods mentioned in Section 2.2 deviate from .05. Since an empirical type‐I error rate close to .05 is preferred, we consider an empirical type‐I error rate between [.04,.06] to be acceptable. For a given scenario, if no confidence interval method yields an empirical type‐I error rate within [.04,.06], the method closest to and below .05 will be preferred.

Results of our simulation study suggest that the type‐I error rate deviates further from the nominal rate of .05 for most confidence interval methods as $\rho_{0}=\rho$ increases. Therefore, in the following sections, we focus on the results for ρ=.9, which represents the scenario with the worst performance. Figures 1, 2, 3, 4 show the heatmaps for different scenarios with ρ=.9. The heatmaps obtained with the other settings of $\rho_{0}=\rho$ can be found in File S1, while complete results can be found in File S2. In the figures, each cell is colour graded based on how far the empirical type‐I error rate deviates from the nominal type‐I error rate of .05 for the specified scenario. In addition, an empirical type‐I error rate greater than .05 is marked with a plus sign (+).

**FIGURE 1:**
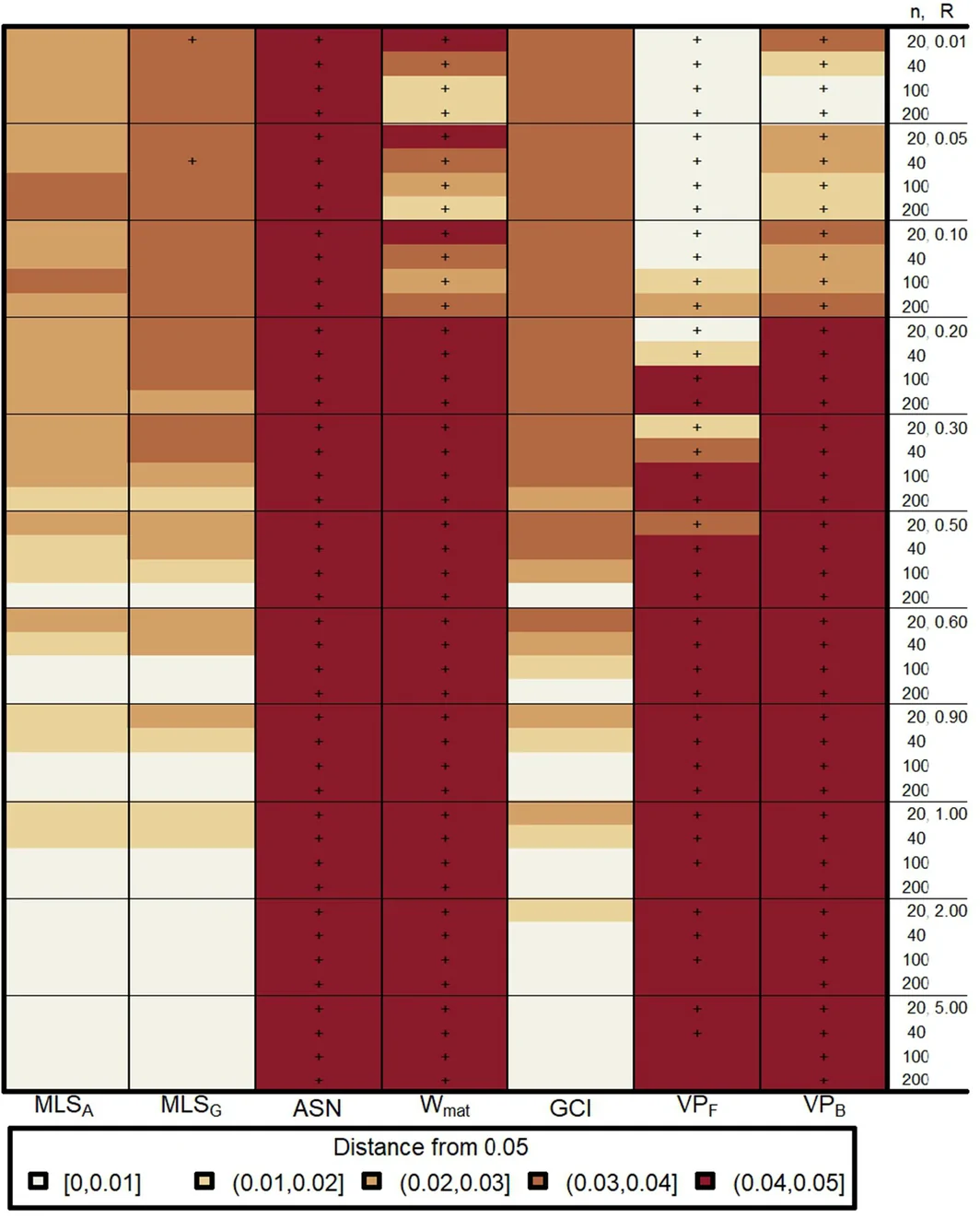
Empirical type‐I error rate for a one‐sided test at α=.05, ρ=.9, k=2 and R∈{.01,.05,.1,.2,.3,.5,.6,.9,1,2,5}. Distances from .05 are colour‐graded; ‘+’ indicates rates exceeding the nominal level.

### 3.2 Results of the simulation study for k=2

We start by presenting the results when there are two raters, that is, k=2 and the rater‐to‐error variance ratio is R<1. Under these settings, Figure 1 shows that $VP_{F}$ provides an empirical type‐I error rate close to the nominal level, since it is within the range [.05,.06] when n≥20 and R≤.05, and $VP_{B}$ when n≥100 and R=.01. The empirical type‐I error rate of these methods moves further away from .05 as R increases. In contrast, the patterns of the empirical type‐I error rate for $MLS_{A}$, $MLS_{G}$ and GCI differ from those of $VP_{F}$ or $VP_{B}$. The type‐I error rates for $MLS_{A}$, $MLS_{G}$ and GCI are farther away from .05 for small values of R, for example, when R≤.3, but move closer to .05 as R increases. When R=.9, $MLS_{A}$, $MLS_{G}$ and GCI provide an empirical type‐I error rate within [.04,.05] only when n≥100, but within [.03,.05] when n>40. When R increases beyond 1 (R>1), the performance of these three methods further improves, and they provide acceptable empirical type‐I error rates for almost all values of n starting from n=20. Figure 1 further shows that when k=2, the empirical type‐I error rates of ASN and $W_{mat}$ are far from .05. For ASN, the empirical type‐I error rate can be as high as .44, while for $W_{mat}$ it can reach .35 (for details, see File S2).

Table 2 summarizes the recommended confidence interval methods under different scenarios studied for R, when k=2, with recommendations based on the worst case scenario, that is, when ρ=.9. When R≤.1, $VP_{F}$ is the best performing confidence interval method in terms of empirical type‐I error rate, while for R>.1, $MLS_{A}$, $MLS_{G}$ and GCI are reasonable options.

**TABLE 2:** Recommended confidence interval methods under different settings of R when k=2.

R setting (with k=2)	Recommended confidence interval methods
R≤.1	$VP_{F}$
.1<R≤.9	$MLS_{A}$
.9<R≤1.0	$MLS_{A}$, $MLS_{G}$
R>1.0	$MLS_{A}$, $MLS_{G}$, GCI

### 3.3 Results of the simulation study for k>2

Figures 2 and 3 present results for R<1 while Figure 4 presents results for R≥1. Under these settings, it can be seen that $VP_{F}$ provides an empirical type‐I error rate within acceptable limits when n≥40, k≤4 and R≤.1, while its performance worsens as R, or k increases. $VP_{B}$ provides an empirical type‐I error rate within acceptable limits when n≥20, k>3 and R≤.1. Similar to $VP_{F}$, the performance of $VP_{B}$ worsens as R increases; however, unlike $VP_{F}$, it improves as k increases. For $W_{mat}$, the type‐I error rate is within [.05,.06] when n≥40, k≥8 and R≤.5. Its performance behaves similarly to $VP_{B}$ with respect to increasing R and k. ASN performs the worst while providing a type‐I error rate within [.05,.06] only in a few scenarios. When R≥1, Figure 4 shows that the type‐I error rate of $VP_{F}$, $VP_{B}$, ASN and $W_{mat}$ worsens, and only $W_{mat}$ provides an acceptable empirical type‐I error rate when k≥10. As for the performance of the remaining methods, the type‐I error rate of $MLS_{A}$, $MLS_{G}$ and GCI show a non‐linearity with respect to R. An increase in R from .01 to .2, results in a worsening performance of their type‐I error rate, but their performance starts to improve when R increases beyond .3. When R=.3, $MLS_{G}$ provides an acceptable type‐I error rate when k≥8, and GCI when k≥15, while the type‐I error rate for $MLS_{A}$ is not within acceptable limits. When R increases beyond 1, it can be observed from Figure 4 that $MLS_{G}$ and GCI provide a type‐I error rate within acceptable limits in almost all scenarios studied, while $MLS_{A}$ provides a type‐I error rate within acceptable limits when R≥2.

**FIGURE 2:**
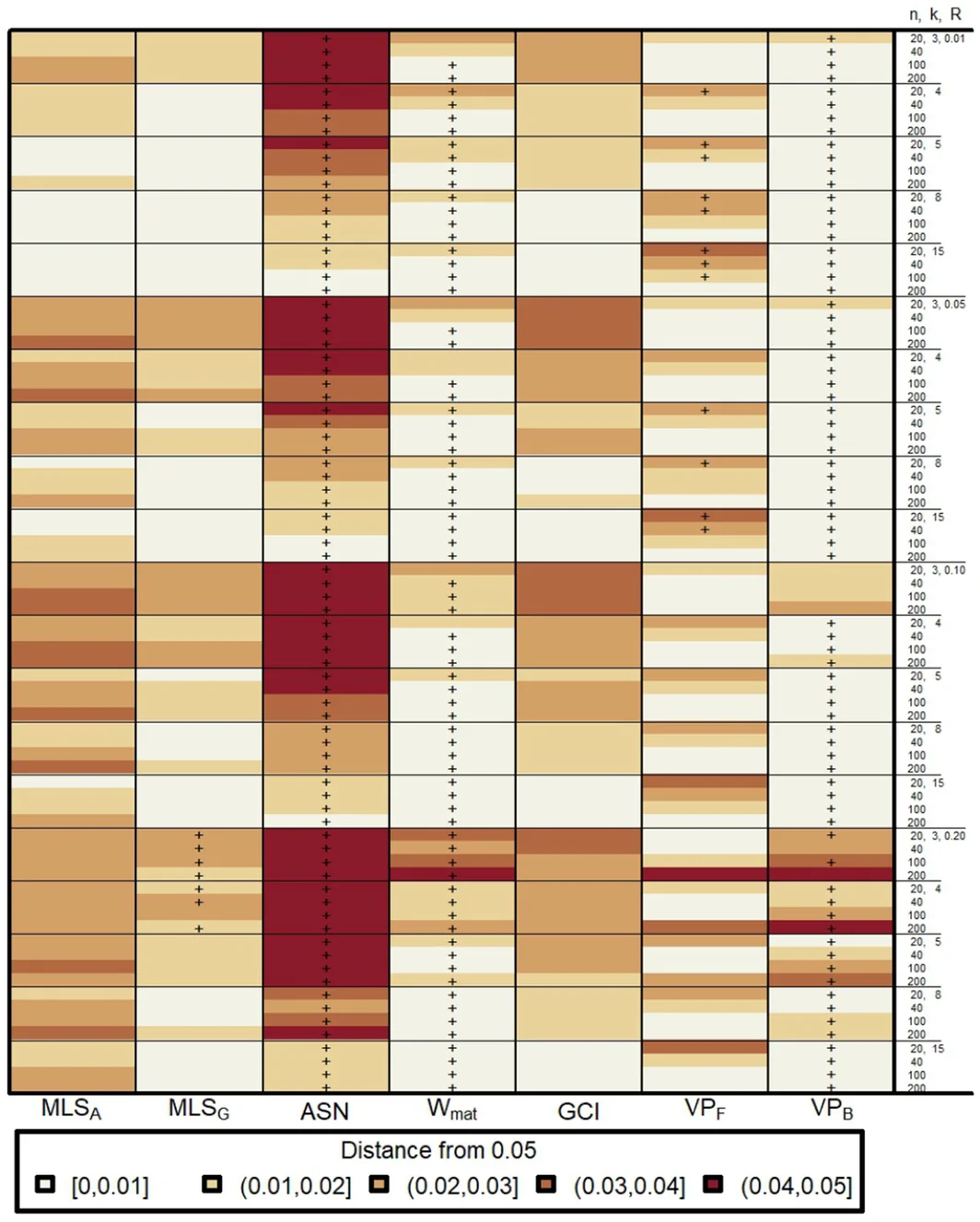
Empirical type‐I error rate for a one‐sided test at α=.05, ρ=.9, k∈{3,4,5,8,15} and R∈{.01,.05,.1,.2}. Distances from .05 are colour‐graded; ‘+’ indicates rates exceeding the nominal level.

**FIGURE 3:**
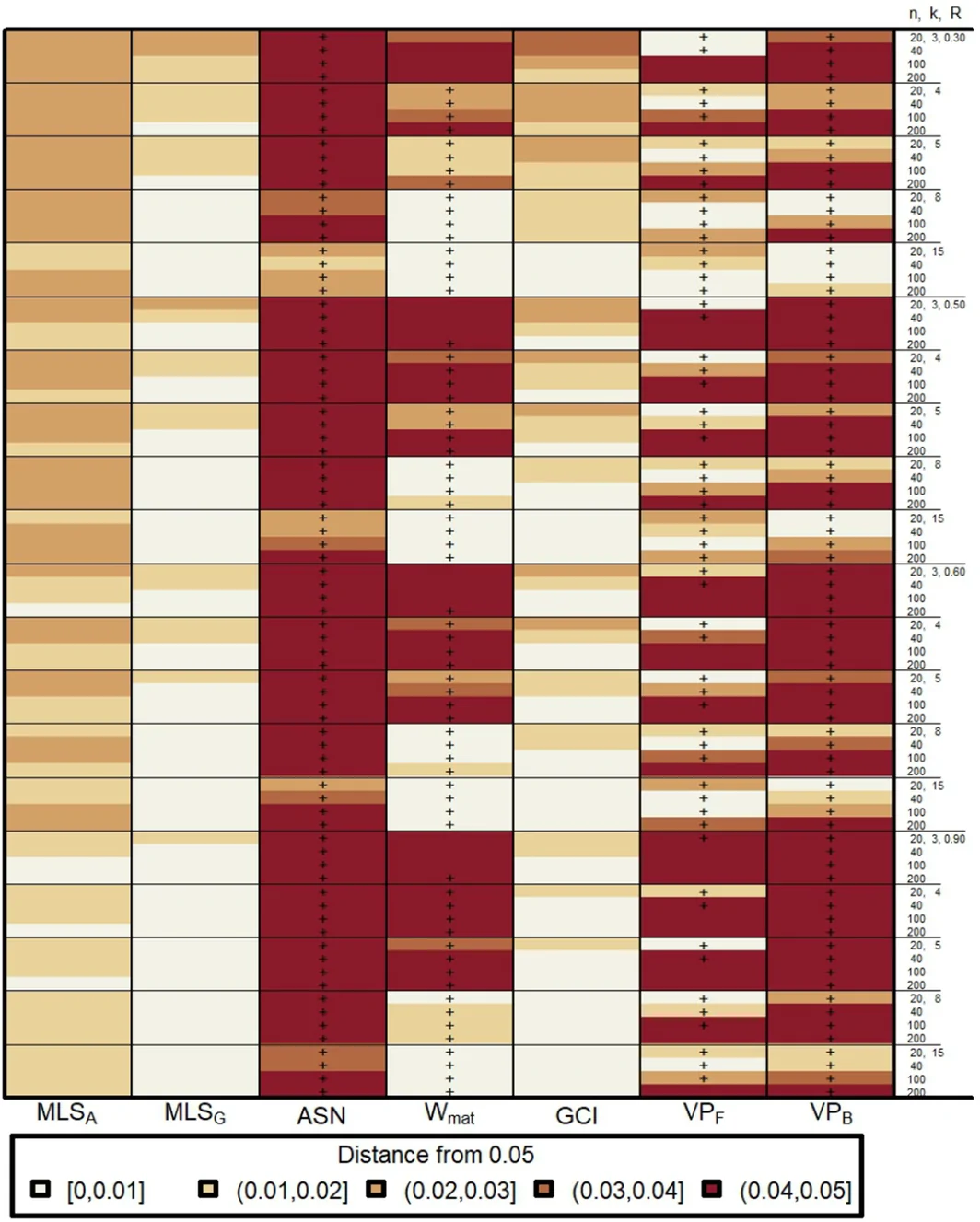
Empirical type‐I error rate for a one‐sided test at α=.05, ρ=.9, k∈{3,4,5,8,15} and R∈{.3,.5,.6,.9}. Distances from .05 are colour‐graded; ‘+’ indicates rates exceeding the nominal level.

**FIGURE 4:**
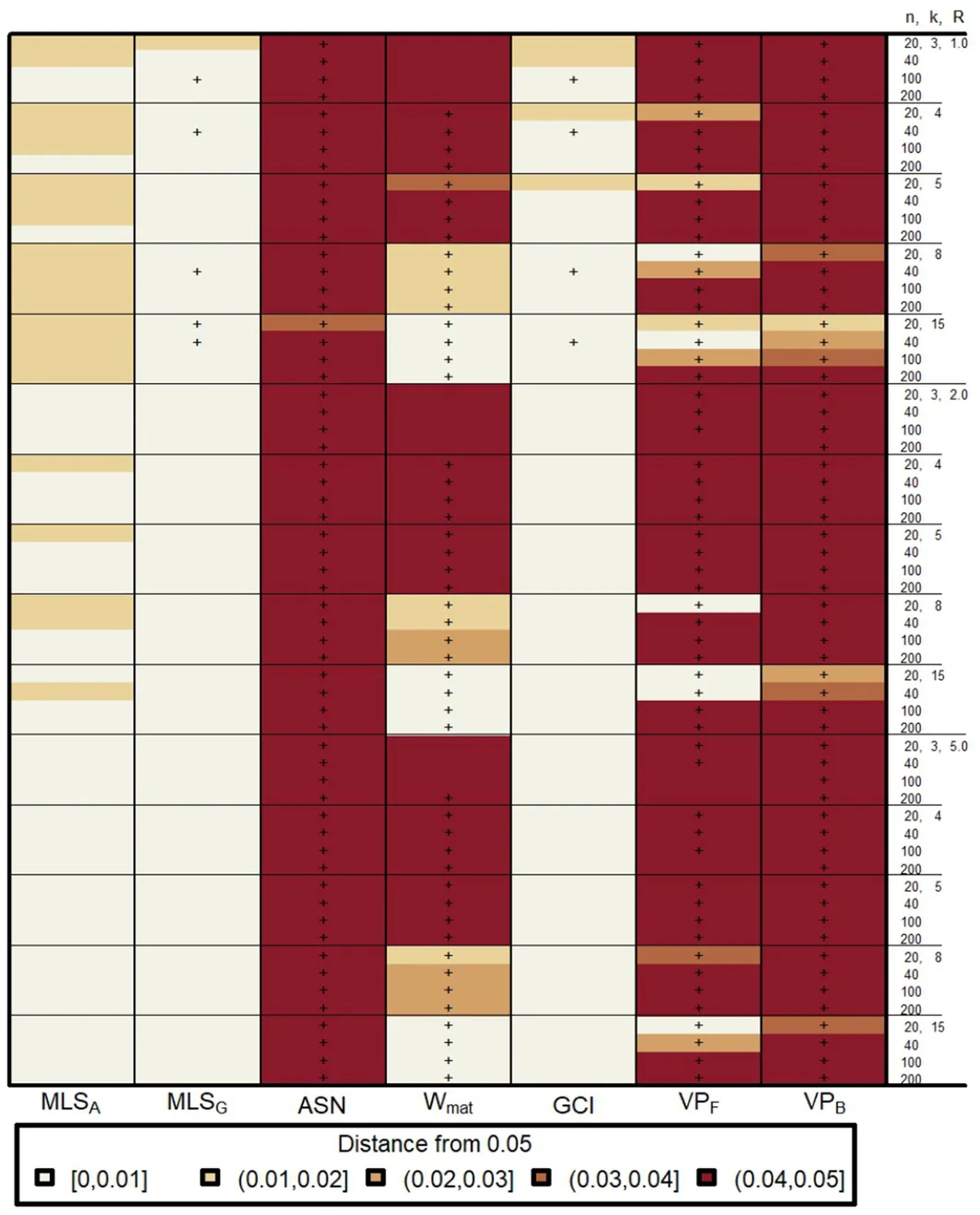
Empirical type‐I error rate for a one‐sided test at α=.05, ρ=.9, k∈{3,4,5,8,15} and R∈{1,2,5}. Distances from .05 are colour‐graded; ‘+’ indicates rates exceeding the nominal level.

Table 3 summarizes the recommended confidence interval methods under different scenarios studied for R, and n when k>2, with recommendations based on the worst case scenario, that is, when ρ=.9. When R≤.1, $VP_{B}$ is the best performing method. $W_{mat}$ provides an acceptable empirical type‐I error rate when k≥8 and R≤.2. Generally, when R≥.2, $MLS_{A}$, $MLS_{G}$ and GCI are reasonable options. The empirical type‐I error rate of these three methods improves as R increases.

**TABLE 3:** Recommended confidence interval methods under different settings of R when k>2.

R setting	k setting	Recommended confidence interval methods
R≤.1	2<k≤4	$VP_{B}$, $VP_{F}$ (n≥40), $W_{mat}$ (n≥100)
	k≥5	$VP_{B}$, $W_{mat}$ (n≥40)
.1<R≤.2	2<k≤4	$MLS_{A}$
	4<k≤8	$MLS_{G}$
	k≥8	$MLS_{G}$, $W_{mat}$
.2<R≤.6	2<k≤4	$MLS_{G}$
	k≥5	$MLS_{G}$, GCI
R>.6	k>2	$MLS_{A}$, $MLS_{G}$, GCI

## 4 SAMPLE SIZE DETERMINATION PROCEDURES

Our interest lies in calculating the minimum number of participants, n satisfying the criterion in Equation (4) given the number of raters, k, the rater‐to‐error variance ratio, R, the type‐I error rate, α and the values of ρ under the null and alternative hypotheses, $\rho_{0}$ and $\rho_{A}$. This ensures that the hypothesis test described in Section 2.1 achieves sufficient power, 1−β. The lower limit of the confidence interval for ρ in Equation (4), L, can be obtained using the different methods described in Section 2.2. The procedure to obtain the minimum sample size required for a study involves finding the power $𝒫(n)=P[L>\rho_{0}|\rho =\rho_{A}]$ for different values of the number of participants, n and thus the minimum n such that 𝒫(n)≥1−β. The procedure to obtain such power function 𝒫(n) is described in Section 4.1. A closed form expression for the sample size following the ASN method is given by Bourredjem et al. (2024) as shown in Appendix A. Since the results of our simulation study in Section 3 showed that ASN does not perform well, we do not consider this method for sample size determination.

### 4.1 Procedures to obtain the power function

The power function depends on the lower limit of the confidence interval for ρ, and is therefore dependent on the method used to construct the confidence interval. In the following subsections, we provide an expression for the power functions using the confidence interval methods $W_{mat}$ (Section 2.2.1) and $VP_{F}$ (Section 2.2.5). We have derived the corresponding expressions in Appendices Dand E. A closed form expression for the power function could not be derived for $VP_{B}$ (Section 2.2.6), $MLS_{A}$ (Section 2.2.2), $MLS_{G}$ (Section 2.2.3) and GCI (Section 2.2.4). For these four methods, we use Monte Carlo simulations to obtain power.

#### 4.1.1 Power function for the confidence interval based on the delta method with matrix formulation ($W_{mat}$)

The power function in this case is given by (see Appendix D for the derivation),

(16) $$𝒫(n)=T_{n+1}\left(\frac{\rho_{A}-\rho_{0}}{\sqrt{V(\hat{\rho }|\rho =\rho_{A})}}-T_{n+1}(1-\alpha )\right).$$

The expression for the estimate of $V(\hat{\rho })$ is given in Equation (6). The expression for $V(\hat{\rho }|\rho =\rho_{A})$ in Equation (16), requires expressing $1^{\prime }S1$, $1^{\prime }S^{2}1$, trS, $trS^{2}$ and $y_{d}^{\prime }y_{d}$ in terms of the parameters $\sigma_{s}^{2}$, $\sigma_{r}^{2}$ and $\sigma_{e}^{2}$. When $\rho =\rho_{A}$, the variance components are replaced by their respective parameter values to obtain a value for the power (for details, see Appendices B, C, D).

#### 4.1.2 Power function for the variance partitioning confidence interval using an F‐distribution ($VP_{F}$)

The power function in this case is given by (see Appendix E for the derivation),

(17) $$𝒫(n)=F_{{df}_{2},{df}_{1}}\left(\frac{\tau (\rho_{0})}{\tau (\rho_{A})}F_{{df}_{1},{df}_{2}}(\alpha )\right),$$

where $\tau (\rho_{0})=\frac{1}{\rho_{0}}-1$, $\tau (\rho_{A})=\frac{1}{\rho_{A}}-1$, $df_{1}=\frac{2\sigma_{s}^{4}}{V({\hat{\sigma }}_{s}^{2})}$ and $df_{2}=\frac{2{\left(\sigma_{r}^{2}+\sigma_{e}^{2}\right)}^{2}}{V({\hat{\sigma }}_{r}^{2})+V({\hat{\sigma }}_{e}^{2})}$. Note that the covariance between ${\hat{\sigma }}_{r}^{2}$ and ${\hat{\sigma }}_{e}^{2}$ is set to 0, following the works of van den Heuvel et al. (Heuvel, 2010), and Demetrashvili et al. (Demetrashvili et al., 2016), who suggested that $VP_{F}$ possesses better statistical properties when the covariance terms are not included. To obtain a value for the power, we substitute the variance of the variance estimators from Appendix B, where the variance components are replaced by their respective parameter values such that $\rho =\rho_{A}$.

#### 4.1.3 Power function for the variance partitioning confidence interval using a beta‐distribution ($VP_{B}$) and the modified large sample confidence intervals ($MLS_{A}$ and $MLS_{G}$)

A closed form expression for the power function is difficult to derive when the confidence interval is obtained using $VP_{B}$, $MLS_{A}$ or $MLS_{G}$. The power function in this case can be obtained using Monte Carlo simulations. The steps for calculating the power function 𝒫(n) for a given n are briefly described below.

Step 1: For given k, $\rho_{A}$, R and n, obtain $10^{4}$ simulated datasets following the procedure described in Section 3.
Step 2: The lower limit of the confidence interval is obtained from Equation (14) (for $VP_{B}$), Equation (7) (for $MLS_{A}$) or Equation (9) (for $MLS_{G}$), for each of the $10^{4}$ simulations, and the power is calculated as the proportion of simulations in which L is greater than $\rho_{0}$.

#### 4.1.4 Power function for the generalized confidence interval (GCI)

As in the case for $VP_{B}$, $MLS_{A}$ and $MLS_{G}$, a closed form expression for the power function is not available for GCI. One can obtain the power using Monte Carlo simulations as described in Section 4.1.3, where in Step 2 the lower limit of the confidence interval is obtained using empirical quantiles of the pivotal quantity in Equation (10) (see Section 2.2.4) which itself requires extensive simulations. Obtaining 𝒫(n) in this way involves a high computational burden and associated long running time. The procedure can however be simplified using a variance‐reduction technique termed ‘inverse Rao‐Blackwellization’ in Dobbin and Ionan (Dobbin & Ionan, 2015). This procedure reduces the number of simulations to obtain the lower limit of the confidence interval, L and involves replacing the estimator Ĝ(α) by,

(18) $${\hat{G}}_{RB}(\alpha )=inf\left\{g:\alpha \leq \frac{1}{M}\sum_{m=1}^{M}P\left[g(t)\leq g|{\left(\chi_{n-1}^{2}\right)}_{m},{\left(\chi_{\left(n-1)(k-1\right)}^{2}\right)}_{m}\right]\right\},$$

where ${\left(\chi_{n-1}^{2}\right)}_{m},{\left(\chi_{\left(n-1)(k-1\right)}^{2}\right)}_{m}$ are $m^{th}$ Monte Carlo observations of $\chi^{2}$ variables, required to estimate MSS and MSE. The lower limit of the confidence interval, which is the conditional probability in Equation (18), can then be directly obtained from quantiles of an inverted gamma distribution using far fewer simulations (say 100 simulations). Therefore, 𝒫(n) can be obtained following the procedure mentioned in Section 4.1.3 where in Step 2, the lower limit of the confidence interval is obtained using Equation (18).

### 4.2 Sample size algorithm and software

The aim of the sample size algorithm is to determine the minimum number of participants, n given k, R, $\rho_{0}$, $\rho_{A}$ and α such that the power for that n, 𝒫(n)≥1−β for a pre‐defined target power 1−β following the methods in Section 4.1. Given k, R, $\rho_{0}$, $\rho_{A}$ and α, 𝒫(n) is generally non‐decreasing in n. File S4 reports evaluations of 𝒫(n) as a function of n across different combinations of k, R, $\rho_{0}$, $\rho_{A}$, illustrating this behaviour. The schematic diagram in Figure 5 then illustrates the steps used to determine the smallest number of participants, $n^{*}$, such that $P(n^{*})\geq 1-\beta$.

**FIGURE 5:**
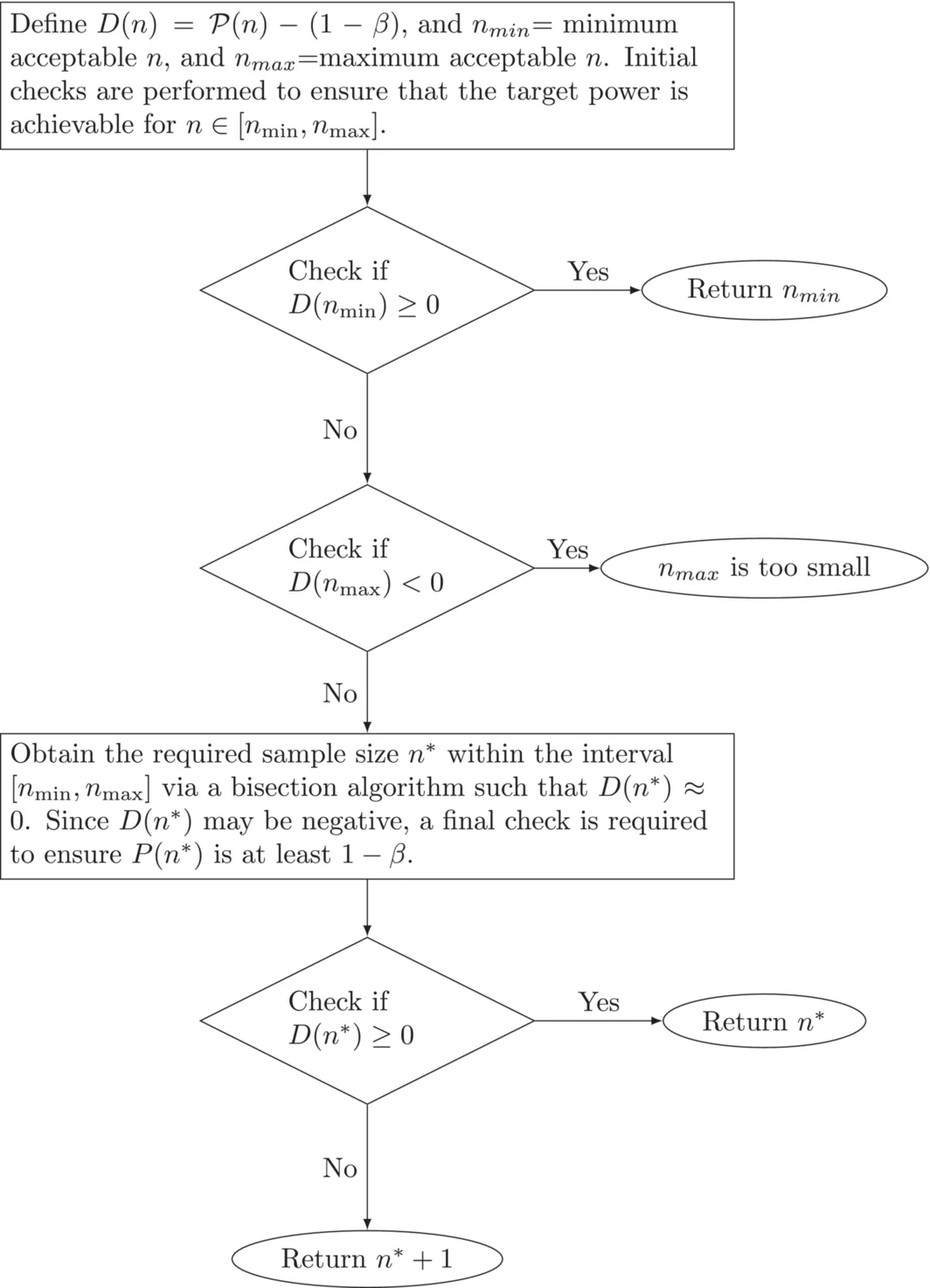
The schematic diagram shows the steps of the sample size determination algorithm to find the minimum number of participants, n to achieve a power of at least 1−β given the number of raters, k, the value for the ICC for agreement under the null hypothesis, $\rho_{0}$ and alternative hypothesis, $\rho_{A}$, and for a given type‐I error rate, α.

Specifically, we first check whether the target power can be achieved within a predefined range of feasible number of participants, $n\in [n_{\min},n_{\max}]$. These initial checks ensure that the bracketing interval $\left[n_{min},n_{max}\right]$ contains a value for n for which the power is equal to or greater than 1−β. Defining an objective function D(n)=𝒫(n)−(1−β), the goal is to find the smallest sample size $n^{*}$, such that $D(n^{*})\approx 0$. To achieve this, we implement a modified bisection algorithm, and the steps of the algorithm are as follows:

1. Set $a=n_{\min}$ and $b=n_{\max}$.
2. Repeat until convergence:
  - Compute $n_{\text{mid}}=\text{Integer}((a+b)/2)$.
  - Evaluate $D(n_{\text{mid}})$.
  - If $D(a)\cdot D(n_{\text{mid}})<0$, update $b\leftarrow n_{\text{mid}}$, otherwise $a\leftarrow n_{mid}$.
  - Stop if |b−a|<1 or $n_{\text{mid}}$ has appeared in a previous iteration.

Upon completion of the algorithm, we obtain $n^{*}$ such that $D(n^{*})\approx 0$. A final check is then performed to ensure that $D(n^{*})\geq 0$, that is, $P(n^{*})\geq 1-\beta$. If the condition is satisifed, $n^{*}$ is returned as the required sample size otherwise, $n^{*}+1$ is returned.

We developed a shiny app to implement the sample size determination procedures described in this paper which is freely available on Github and is hosted online at shinyApps. The app enables researchers to obtain a sample size for a reliability study via an interactive and easy‐to‐use graphical interface.

## 5 EVALUATION OF SAMPLE SIZE DETERMINATION PROCEDURES

Sample sizes are calculated using the procedures in Section 4. We then assess whether the resulting sample sizes provide sufficient empirical power and adequately control the empirical type‐I error rate. This is done using the Monte Carlo setup described in Section 3. Specifically, we conduct two separate sets of Monte Carlo simulations, using $10^{5}$ datasets, at the calculated sample size. The first set is done under $\rho =\rho_{A}$ to estimate power, and the second under $\rho =\rho_{0}$ to estimate the type‐I error rate. We consider an empirical power above or equal to .79 and a type‐I error rate below or equal to .06 acceptable, allowing a distance of .01 from the target power of .80 and from the nominal type‐I error rate of .05, respectively.

Table 4 shows sample sizes obtained using the procedures in Section 4 for R=.1, k∈{2,3,5,10,15}, α=.05 and different values of $\rho_{A}$ and $\rho_{0}$, to achieve a minimum power of 1−β=.8. Since according to Section 3, we recommend using the methods $VP_{F}$, $VP_{B}$ or $W_{mat}$ when R≤.1, sample sizes are calculated using these methods. As expected, as the difference between $\rho_{A}$ and $\rho_{0}$ decreases, a larger sample size for n is required to achieve a power .8 for the hypothesis test. Conversely, as k increases, a smaller sample size for n is required to achieve the same power. It can be also observed that, for a given scenario, the calculated sample sizes using $VP_{F}$ and $VP_{B}$ are rather close, while those of $W_{mat}$ are substantially higher, though this difference reduces as k increases. Furthermore, in some scenarios, a sample size of less than 1000 was insufficient to achieve a power of .8 using $W_{mat}$. Consequently, the corresponding cells in the table are left empty. This is probably due to the power of the hypothesis test approaching a limit below .8 as n→∞ under these scenarios.

**TABLE 4:** Minimum number of participants, n, for different settings of $\rho_{0}$ and $\rho_{A}$, fixing α=.05, k∈{2,3,5,10,15} and R=.1, to achieve a power of 1−β=.8 according to $VP_{F}$, $VP_{B}$ and $W_{mat}$. Blank cells indicate that n=1000 was insufficient to achieve 80% power; bold entries denote sample sizes with empirical power <.79, and ‘∗’ indicates empirical type‐I error rate greater than .06.

*ρ*_*A*_	*ρ*_*0*_	*k*	*VP*_*F*_ *(n)*	*VP*_*B*_ *(n)*	*W*_*mat*_ *(n)*
.8	.5	2	18*	20*	68*
		3	**15**	14*	23
		5	14	12	16
		10	13	10	13
		15	12	10	12
.8	.6	2	35*	41*	
		3	**28**	26	54
		5	24	20	28
		10	22	17	22
		15	21	16	20
.8	.7	2	139*	177*	
		3	**91**	97*	
		5	72	67	127
		10	62	55	62
		15	59	51	55
.9	.6	2	11	10*	29*
		3	10	8*	18*
		5	9	7	14
		10	9	6	12
		15	9	6	12
.9	.7	2	18	16*	57*
		3	15	12*	30*
		5	14	10	23
		10	13	9	19
		15	13	9	18
.9	.8	2	48*	49*	
		3	**37**	33*	106*
		5	32	26	54
		10	29	23	43
		15	28	22	40

Having calculated the sample size, we evaluated the empirical power and the type‐I error rate using simulations. The sample sizes showing an empirical power smaller than .79 are marked in bold, while those for which the empirical type‐I error rate exceeds .06 are indicated with an asterisk (∗). Empirical values for power and type‐I error rate are reported in File S3. It can be seen from Table 4 that sample sizes obtained using $VP_{B}$ and $W_{mat}$ yield an empirical power greater than .79 under all scenarios studied. As for $VP_{F}$, an empirical power greater than .79 is obtained when k≥5 under all scenarios. As for the empirical type‐I error rate, Table 4 also shows that when the number of raters is small, that is, k≤3, the type‐I error rate was not sufficiently controlled, with several instances showing type‐I error rates beyond .06.

Table 5 shows sample sizes obtained using the procedures in Section 4 for R∈{.5,1,2}, k∈{2,3,5,10,15}, α=.05 and different values of $\rho_{A}$ and $\rho_{0}$, to achieve a minimum power of 1−β=.8. Since according to Section 3, under these settings, we recommend using $MLS_{A}$, $MLS_{G}$ or GCI, sample sizes are calculated using these methods. Scenarios where a sample size <1000 was not enough to achieve a minimum power level of .8 for each of the three confidence interval methods, are removed. This includes all scenarios where k=2 and k=3. We observe patterns for the minimum number of participants n similar to Table 4 with respect to the differences between $\rho_{A}$ and $\rho_{0}$ as well as with respect to k. It can be observed in Table 5 that as R increases, a larger sample size for n is required to achieve the same power. The three methods show sample sizes which are generally close. The empirical power obtained using simulations was greater than .79 and the empirical type‐I error rate was below .06 for all the scenarios and all three confidence interval methods in Table 5 (see File S3).

**TABLE 5:** Minimum number of participants, n, for different settings of $\rho_{0}$ and $\rho_{A}$, fixing α=.05, k∈{5,10,15} and R∈{.5,1,2} to achieve a power of 1−β=.8 according to $MLS_{A}$, $MLS_{G}$ and GCI. Combinations where n=1000 were insufficient to achieve 80% power have been omitted. All displayed sample sizes provide sufficient empirical power (≥.79) and sufficient control of the empirical type‐I error rate (≤.06).

*ρ*_*A*_	*ρ*_*0*_	*R*	*k*	*MLS*_*A*_ *(n)*	*MLS*_*G*_ *(n)*	*GCI (n)*
.8	.5	.5	5	69	42	67
			10	12	10	12
			15	10	9	10
		1	10	14	12	14
			15	11	10	11
		2	10	17	16	17
			15	12	11	12
.8	.6	.5	10	33	26	30
			15	22	18	21
		1	10	83	53	73
			15	28	23	25
		2	15	43	34	41
.8	.7	.5	15	344	156	257
.9	.6	.5	5	12	10	13
			10	7	6	7
			15	6	6	6
		1	5	22	17	23
			10	7	7	8
			15	6	6	6
		2	10	8	7	9
			15	6	6	7
.9	.7	.5	5	91	51	111
			10	12	11	12
			15	10	9	10
		1	10	15	13	15
			15	11	10	11
		2	10	19	17	19
			15	12	11	13
.9	.8	.5	10	84	52	62
			15	37	29	34
		1	15	66	47	59
		2	15	411	243	961

## 6 PRACTICAL GUIDANCE FOR SAMPLE SIZE DETERMINATION

Based on the evaluation of the sample size procedures presented in Tables 4and 5, we provide the following guidance for selecting parameter values for $\rho_{A}$ and R when planning a reliability study. Researchers are advised to specify plausible ranges for these quantities and adopt a min–max strategy: find the smallest sample size satisfying the required power under the worst‐case combination of the parameter values. Given a type‐I error rate α, a desired power level 1−β, and an acceptable lower bound for the ICC, $\rho_{0}$, the following principles support conservative sample size planning:

1. Given $\rho_{A}$ and k, a larger value of R generally increases the required sample size of participants, n. Choosing the upper bound of plausible values for R is therefore conservative (the worst‐case scenario).
2. Given R and k, as the difference between $\rho_{A}$ and $\rho_{0}$ decreases, the required sample size increases. Choosing $\rho_{A}$ close to $\rho_{0}$ therefore represents the worst‐case scenario for $\rho_{A}$, yielding a conservative design.

Note that for certain combinations of $\rho_{0}$ and $\rho_{A}$, a feasible sample size (e.g. below 1000 participants) may not be attainable for specific settings of R and k. This becomes particularly relevant for large values of R and small values of k. While the estimated R in several reliability studies is less than one (Hernandez Engelhart et al., 2024; Bland & Altman, 1999; Cutti et al., 2010; Dobbin & Ionan, 2015; Fleiss & Shrout, 1978; Hart, 2019; Streiner & Norman, 1995; Ting et al., 2009), R depends on the study design and the rater population and can hardly be modified. If it is practically feasible, one solution is to increase the number of raters (k) to obtain a feasible sample size. Although sample size procedures can be applied with as few as two raters, Briesch et al. (2014) and ten Hove et al. (Hove et al., 2019) recommend using at least three raters. Our evaluation of the type‐I error rate and power in Tables 4 and 5 suggests that at least five raters are needed for the proposed procedures to perform adequately.

## 7 EMPIRICAL ILLUSTRATION

In this section, we illustrate how the confidence interval methods in Section 2.2 and the sample size determination procedures in Section 4 can be used in a practical situation. We use the study by Engelhart et al. (Hernandez Engelhart et al., 2024) for illustration. The study focuses on fetal heart rate (FHR) monitoring in an obstetric setting. In clinical practice, midwives use a handheld Doppler device to measure FHR during intermittent auscultation. If abnormalities, such as accelerations or decelerations from the baseline heart rate, are detected, intervention decisions need to be made. Inter‐rater agreement is therefore important in this context, as disagreements may lead to different interventions for the mother and the fetus. The primary aim of the study by Engelhart et al. (Hernandez Engelhart et al., 2024) was therefore to evaluate the inter‐rater agreement of fetal heart rates measured using a handheld Doppler device. The FHR was determined from one minute recordings (beats per minute, bpm) on the same n=154 women undergoing labour by k=16 midwives. After removing missing values, data from 145 mothers by 16 midwives remained.

The data from the study can be suitably modelled using a two‐way ANOVA model (see Equation (1)) which assumes the normality of the FHR measurements of each midwife and for each fetus, and the homoscedasticity of the variance of the measurements between midwives and between fetuses. Figure 6 summarizes the FHRs given by the 16 midwives. Exploratory analysis revealed that the absolute value of skewness is less than 1 and the value of the excess kurtosis is less than 2 (averaged across midwives), which implies that measurements can be considered to be normally distributed (Curran et al., 1996; Hair et al., 2019). Mild heteroscedasticity between midwives was observed. The ICC for agreement was estimated as $\hat{\rho }=.735\;$ (95% confidence interval according to GCI: (.691,1)), and the rater‐to‐error variance ratio as $\hat{R}=.09$. The value of the ICC for agreement indicates that 73.5% of the FHR variability can be attributed to variability between the fetuses. The confidence interval for the ICC for agreement obtained using the seven confidence interval methods are given in Table 6. The reliability was considered to be sufficient to monitor FHR using a handheld Doppler device (Hernandez Engelhart et al., 2024).

**FIGURE 6:**
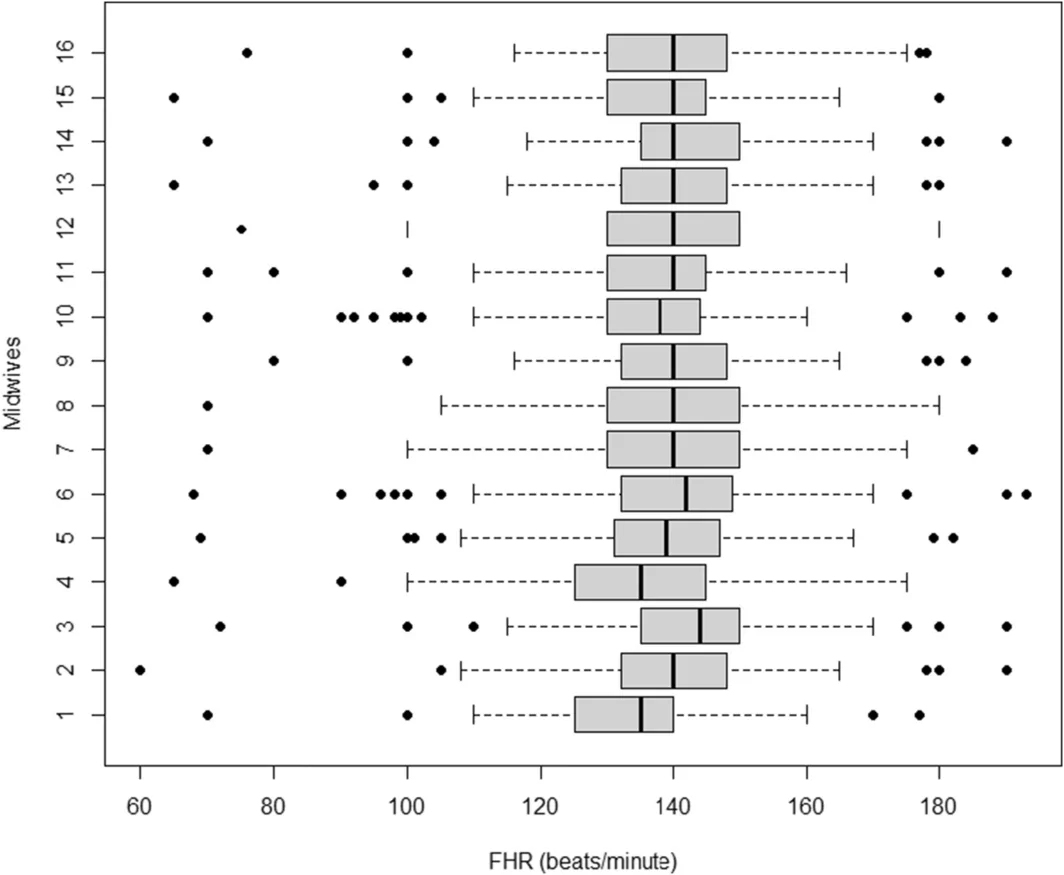
Boxplots of fetal heart measurements of 145 mothers made by 16 midwives using a handheld Doppler device.

**TABLE 6:** Lower limit of the 95% one‐sided confidence intervals for the ICC for agreement using the seven confidence interval methods. The upper limit of the confidence intervals is 1.

*MLS*_*A*_	*MLS*_*G*_	*ASN*	*W*_*mat*_	*GCI*	*VP*_*F*_	*VP*_*B*_
.683	.693	.689	.692	.691	.690	.693

It can be seen from Table 6 that the lower limit of the confidence intervals, except for $MLS_{A}$, are rather close across the different methods. Considering that the ICC for agreement for the population of fetuses and midwives is around .7, and the corresponding rater‐to‐error variance ratio is around .1, we deduce that all the methods except $MLS_{A}$ are likely to yield a type‐I error rate close to .05 (see Figure 2 in File S1). This also aligns with Table 3, which shows that $MLS_{A}$ does not provide an acceptable empirical type‐I error rate when R≤.2 and k≥8.

To demonstrate the practical application of the sample size determination procedures in Section 4, we consider a scenario where researchers are planning a reliability study for the measurement of FHR (bpm) using a handheld Doppler device. Suppose that the researchers anticipate an ICC for agreement to be .8. They want to obtain the sample size for the number of participants to achieve a power of .8 to statistically test if the ICC for agreement is greater than .7. Further, suppose that they expect the rater‐to‐error variance ratio, R, to be .1 and the maximum number of raters k available for the study is 15. Employing the sample size determination procedures in Section 4, they can obtain the minimum number of participants under a pre‐specified rater‐to‐error variance ratio, R and the number of raters, k. Table 7 shows sample sizes for R=.1 and k∈{10,15}. Sample sizes are obtained using the recommended confidence interval methods, $VP_{B}$ or $W_{mat}$ under the given scenarios (for details, see Section 3.3).

**TABLE 7:** Minimum number of participants needed for testing the statistical hypothesis $H_{0}:\rho_{0}=.7$ vs $H_{a}:\rho_{A}=.8$ with a power of .80, a type‐I error rate α=.05, R=.1 and k∈{10,15}.

*k*	*W*_mat_ (*n*)	*VP*_*B*_ (*n*)
10	62	55
15	55	51

Considering that the cost of hiring raters is generally higher than the cost of recruiting participants for the study, results in Table 7 indicate the benefit of reducing the number of raters outweighs the reduction in the number of participants. Therefore, the researchers can opt for k=10 raters and n=55 participants.

## 8 DISCUSSION

During the planning of a reliability study, researchers often aim to determine the minimum sample size needed to test whether the ICC exceeds an acceptable value. When data is modelled using a two‐way ANOVA model, two ICC coefficients can be estimated, the ICC for agreement and consistency. This paper presents procedures to calculate the minimum sample size for testing the ICC for agreement, based on the lower limit of its one‐sided confidence interval. They were implemented in an R shiny app which provides users with an easy‐to‐use graphical interface to obtain sample sizes.

A literature review identified seven methods for constructing confidence intervals for the ICC for agreement (Mondal et al., 2025). Our Monte Carlo simulation compared these methods based on empirical type‐I error rate, highlighting the importance of the rater‐to‐error variance ratio (R). When R≤.1, $VP_{F}$ performs best when k=2, while $VP_{B}$ for k>2. For R>.1 and k≥2, $MLS_{A}$, $MLS_{G}$ and GCI perform well, while $W_{mat}$ is reasonable only in limited scenarios. ASN did not perform well under most scenarios and was not further studied.

Procedures to determine a sample size for the number of participants, n, were developed to ensure they reach at least a predefined power, 1−β for the hypothesis test given the number of raters, k, the rater‐to‐error variance ratio, R, a type‐I error rate, α and the values of ICC under the null and alternate hypotheses, $\rho_{0}$ and $\rho_{A}$, respectively. Six confidence interval methods, $VP_{F}$, $VP_{B}$, $W_{mat}$, $MLS_{A}$, $MLS_{G}$ and GCI were utilized in determining procedures for sample size calculations. When a closed form expression for the power function could not be derived, we estimated the power using simulations. We found that the obtained sample size provided sufficient empirical power, except in a few of the scenarios studied. Furthermore, we found that a thousand participants were not enough in some cases to obtain the desired power level. This is probably because the empirical power under the specified combination of k, R, α, $\rho_{0}$ and $\rho_{A}$ is smaller than the predefined level of 1−β.

The results of the sample size determination showed that the choice of the confidence interval method impacts the sample size for the number of participants required for the reliability study. Tables 2 and 3 recommend confidence interval methods based on configurations of k and R considering the worst case scenario for ρ (that is, ρ=.9), while Section 4 proposes procedures to determine sample size for hypothesis testing utilizing the recommended confidence interval methods. Researchers are encouraged to carefully assess the specific requirements of their study when selecting a procedure. Section 6 offers guidance on selecting combinations of $\rho_{A}$ and R that yield the smallest required sample size under the worst case combination of the parameter values. Together, these support rigorous planning of reliability studies to ensure sufficient power for testing whether the ICC for agreement exceeds a specified threshold.

Our study is not without limitations. First, the estimator for the ICC for agreement and its corresponding confidence intervals are based on the assumptions of normality of the errors and of the random effects, as well as homoscedasticity of the variances, in line with the two‐way ANOVA model described in Equation (1). Violations of these assumptions can affect the statistical properties of the confidence intervals. Therefore, we recommend that researchers assess these assumptions before applying the procedures described in this paper. In case of violation of the normality assumption, the ICC can be estimated non‐parametrically following the method by Lu et al. (2014) while its confidence interval may be obtained using resampling methods following Feng et al. (2014). However, a sample size determination procedure for these methods is not yet available. Second, our study assumes a complete design in which each rater in the reliability study evaluates all participants. Although Hove et al. (2022) have discussed estimating the ICC for agreement when measurements are missing by design, no procedure to determine sample size has yet been developed in this case either. Third, although our model assumes rater effects to be random, there are settings, for example, when the raters represent a fixed set of items of a questionnaire, where it is more appropriate to consider them as fixed effects. While the point estimation of the ICC for agreement will remain unchanged, the distributional properties of the resulting confidence intervals will differ (Rousson et al., 2003). In particular, the confidence interval is expected to be shorter when the raters are treated as fixed. This is due to the reduced variance of the mean squares between raters, when rater effects are considered fixed (see Appendix F). In the context of our hypothesis testing framework, this implies that the lower bound of the confidence interval is likely to be closer to the point estimate, potentially yielding higher power or a smaller required sample size, depending on the objective. Consequently, the procedures developed in our manuscript under the random effects assumption may be conservative when the rater effect is, in fact, fixed. Investigation of these distributional differences and development of corresponding sample size procedures is left for future research. Fourth, the ANOVA model used in this study does not include an interaction term between participants and raters because each rater made only one rating on every participant. It is therefore not possible to disentangle the interaction effect from the residual error in this design. To incorporate such an interaction, repeated measurements by each rater on each participant would be required. Dobbin and Ionan (2015) examined the impact of ignoring this interaction when using the GCI method and found that it can lead to underestimation of the width of the confidence interval, particularly when the number of participants is small. The extent to which other confidence interval methods are affected by this, and how the power of the hypothesis test is impacted when the rater‐by‐participant interaction is ignored, remains an open question and a possible direction for future research.

## AUTHOR CONTRIBUTIONS

**Dipro Mondal:** conceptualization; methodology; software; formal analysis; visualization; writing – original draft; writing – review and editing. **Alberto Cassese:** conceptualization; methodology; writing – original draft; writing – review and editing; supervision. **Math J. J. M. Candel** and **Sophie Vanbelle:** conceptualization; writing – original draft; writing – review and editing; methodology; funding acquisition; supervision.

## CONFLICT OF INTEREST STATEMENT

The authors declare no conflicts of interest.

## DISCLOSURE OF ARTIFICIAL INTELLIGENCE‐GENERATED CONTENT (AIGC) TOOLS

Not applicable.

## Supporting information


Data S1.

## Data Availability

The data that support the findings of this study are openly available in sample‐size‐hypothesistesting at https://github.com/DiproMondal/sample‐size‐hypothesistesting.

## APPENDIX A Sample size formula for the asymptotically normal confidence interval (ASN)

Following Bourredjem et al. (2024), a sample size formula for the hypothesis test mentioned in Section 4 is,

(19)
$$n=\frac{2{\left(z_{1-\alpha }+z_{1-\beta }\right)}^{2}}{{\left(\rho_{A}-\rho_{0}\right)}^{2}}\rho_{A}^{4}\left({\left(\frac{1}{\rho_{A}}-1\right)}^{2}+c_{nk}\frac{R^{2}{\left(1-\rho_{A}\right)}^{2}}{\rho_{A}^{2}{\left(R+1\right)}^{2}}\right),$$

where $c_{nk}$ is the desired ratio between n and k, $\frac{n}{k}$, for the study design. Note that the sample size expression in Equation (5) in Bourredjem et al. (Bourredjem et al., 2024) is incorrect as it does not contain the square over the expression $\left(\frac{1}{\rho_{A}}-1\right)$.

## APPENDIX B Variance and covariance of the variance component estimates based on the ANOVA model

Note that, following from the assumptions of the ANOVA model in Equation (1), $MSS∼\chi_{n-1}^{2}\frac{k\sigma_{s}^{2}+\sigma_{e}^{2}}{n-1}$, $MSR∼\chi_{k-1}^{2}\frac{n\sigma_{r}^{2}+\sigma_{e}^{2}}{k-1}$ and $MSE∼\chi_{\left(n-1)(k-1\right)}^{2}\frac{\sigma_{e}^{2}}{\left(k-1)(n-1\right)}$ where $\chi_{n-1}^{2}$, $\chi_{k-1}^{2}$ and $\chi_{\left(n-1)(k-1\right)}^{2}$ are a set of jointly independent $\chi^{2}$ random variables, their expected values and variances can be obtained as,

$$\begin{array}{cc} & E(MSS)=k\sigma_{s}^{2}+\sigma_{e}^{2},\;\;V(MSS)=\frac{2{\left(k\sigma_{s}^{2}+\sigma_{e}^{2}\right)}^{2}}{n-1},\\ & E(MSR)=n\sigma_{r}^{2}+\sigma_{e}^{2},\;\;V(MSR)=\frac{2{\left(n\sigma_{r}^{2}+\sigma_{e}^{2}\right)}^{2}}{k-1},\\ & E(MSE)=\sigma_{e}^{2},\;\;V(MSE)=\frac{2\sigma_{e}^{4}}{\left(k-1)(n-1\right)}. \end{array}$$

The variance of the estimator of participant variance is,

$$V({\hat{\sigma }}_{s}^{2})=V\left(\frac{1}{k}(MSS-MSE)\right)=\frac{1}{k^{2}}\left(V(MSS)+V(MSE)\right).$$

The variance of the estimator of rater variance is,

$$V({\hat{\sigma }}_{r}^{2})=V\left(\frac{1}{n}(MSR-MSE)\right)=\frac{1}{n^{2}}\left(V(MSR)+V(MSE)\right).$$

The variance of the estimator of the residual variance is,

$$V({\hat{\sigma }}_{e}^{2})=V(MSE).$$

The covariance between estimator of participant and error variances is,

$$cov({\hat{\sigma }}_{s}^{2},{\hat{\sigma }}_{e}^{2})=cov\left(\frac{1}{k}(MSS-MSE),MSE\right)=-\frac{1}{k}V(MSE).$$

The covariance between estimator of rater and error variances is,

$$cov({\hat{\sigma }}_{r}^{2},{\hat{\sigma }}_{e}^{2})=cov\left(\frac{1}{n}(MSR-MSE),MSE\right)=-\frac{1}{n}V(MSE).$$

The covariance between estimator of participant and rater variances is,

$$cov({\hat{\sigma }}_{s}^{2},{\hat{\sigma }}_{r}^{2})=cov\left(\frac{1}{k}(MSS-MSE),\frac{1}{n}(MSR-MSE)\right)=\frac{1}{nk}V(MSE).$$

## APPENDIX C Expressing the elements of $V(\hat{\rho })$ for the delta method (Equation 6) in terms of the variance components

First, it must be noted that

1. (20)
  $$1^{\prime }S1=kMSS=k^{2}{\hat{\sigma }}_{s}^{2}+k{\hat{\sigma }}_{e}^{2},\text{and}$$
2. (21)
  $$trS=MSS+(k-1)MSE=k({\hat{\sigma }}_{s}^{2}+{\hat{\sigma }}_{e}^{2})$$

### C.0.1 $1^{\prime }S^{2}1$

To derive an expression for $1^{\prime }S^{2}1$ in terms of the variance components we start from a result in Almehrizi and Emam (Page 19 (Almehrizi & Emam, 2021)) that $21^{\prime }S^{2}1$ is an estimate of $n\;cov\left(\frac{n-1}{n}1^{\prime }S1,\frac{n-1}{n}trS\right)$. So, $1^{\prime }S^{2}1$ is an estimate of

(22)
$$\frac{{\left(n-1\right)}^{2}}{2n}cov(1^{\prime }S1,trS).$$

Now substituting the terms $1^{\prime }S1$ and trS by their variance components expression in Equations 20 and 21, the expression in Equation (22) can be elaborated as,

$$\begin{array}{cc} & \frac{{\left(n-1\right)}^{2}}{2n}cov(1^{\prime }S1,trS)\\ & =\frac{{\left(n-1\right)}^{2}}{2n}cov((k^{2}{\hat{\sigma }}_{s}^{2}+k{\hat{\sigma }}_{e}^{2}),k({\hat{\sigma }}_{s}^{2}+{\hat{\sigma }}_{e}^{2}))\\ & =\frac{{\left((n-1)k\right)}^{2}}{2n}cov((k{\hat{\sigma }}_{s}^{2}+{\hat{\sigma }}_{e}^{2}),({\hat{\sigma }}_{s}^{2}+{\hat{\sigma }}_{e}^{2}))\\ & =\frac{{\left((n-1)k\right)}^{2}}{2n}(k\;V({\hat{\sigma }}_{s}^{2})+V({\hat{\sigma }}_{e}^{2})+(k+1)cov({\hat{\sigma }}_{s}^{2},{\hat{\sigma }}_{e}^{2})). \end{array}$$

Therefore, $1^{\prime }S^{2}1$ estimates $\frac{{\left((n-1)k\right)}^{2}}{2n}(k\;V({\hat{\sigma }}_{s}^{2})+V({\hat{\sigma }}_{e}^{2})+(k+1)cov({\hat{\sigma }}_{s}^{2},{\hat{\sigma }}_{e}^{2}))$, where the expressions for $V({\hat{\sigma }}_{s}^{2})$, $V({\hat{\sigma }}_{e}^{2})$ and $cov({\hat{\sigma }}_{s}^{2},{\hat{\sigma }}_{e}^{2}))$ are substituted from Appendix B.

### C.0.2 $trS^{2}$

To derive an expression for $trS^{2}$ in terms of the variance components, we start from a result in Almehrizi and Emam (Page 19 (Almehrizi & Emam, 2021)), that $2trS^{2}$ is an estimate of $nV\left(\frac{n-1}{n}trS\right)$. So $trS^{2}$ is an estimate of

(23)
$$\frac{{\left(n-1\right)}^{2}}{2n}V(trS).$$

Now substituting the expression for trS in Equation (21) and (23) can be elaborated as,

$$\begin{array}{cc} & \frac{{\left(n-1\right)}^{2}}{2n}V(trS)\\ & =\frac{{\left(n-1\right)}^{2}}{2n}V(k({\hat{\sigma }}_{s}^{2}+{\hat{\sigma }}_{e}^{2}))\\ & =\frac{{\left((n-1)k\right)}^{2}}{2n}V({\hat{\sigma }}_{s}^{2}+{\hat{\sigma }}_{e}^{2})\\ & =\frac{{\left((n-1)k\right)}^{2}}{2n}(V({\hat{\sigma }}_{s}^{2})+V({\hat{\sigma }}_{e}^{2})+2cov({\hat{\sigma }}_{s}^{2},{\hat{\sigma }}_{e}^{2})). \end{array}$$

Therefore, $trS^{2}$ estimates $\frac{{\left((n-1)k\right)}^{2}}{2n}(V({\hat{\sigma }}_{s}^{2})+V({\hat{\sigma }}_{e}^{2})+2cov({\hat{\sigma }}_{s}^{2},{\hat{\sigma }}_{e}^{2}))$, where the expressions for $V({\hat{\sigma }}_{s}^{2})$, $V({\hat{\sigma }}_{e}^{2})$ and $2cov({\hat{\sigma }}_{s}^{2},{\hat{\sigma }}_{e}^{2}))$ are substituted from Appendix B.

## APPENDIX D Power function for the delta method with matrix formulation

Substituting the lower limit of the confidence interval (Equation 5) in Equation (4), we obtain,

$$\begin{array}{cc} & P[\hat{\rho }-T_{n+1}(1-\alpha )\sqrt{\hat{V}(\hat{\rho })}>\rho_{0}|\rho =\rho_{A}]\geq 1-\beta ,\\ & \;\Leftrightarrow \;P[\hat{\rho }>\rho_{0}+T_{n+1}(1-\alpha )\sqrt{\hat{V}(\hat{\rho })}|\rho =\rho_{A}]\geq 1-\beta ,\\ & \approx P[\hat{\rho }>\rho_{0}+T_{n+1}(1-\alpha )\sqrt{V(\hat{\rho }|\rho =\rho_{A})}]\geq 1-\beta ,\\ & \;\Leftrightarrow \;P\left[\frac{\rho_{A}-\hat{\rho }}{\sqrt{V(\hat{\rho }|\rho =\rho_{A})}}<\frac{\rho_{A}-\rho_{0}}{\sqrt{V(\hat{\rho }|\rho =\rho_{A})}}-T_{n+1}(1-\alpha )|\rho =\rho_{A}\right]\geq 1-\beta . \end{array}$$

The power function can then be obtained as,

$$T_{n+1}\left(\frac{\rho_{A}-\rho_{0}}{\sqrt{V(\hat{\rho }|\rho =\rho_{A})}}-T_{n+1}(1-\alpha )\right).$$

The expression for $V(\hat{\rho })$ requires that $1^{\prime }S1$, $1^{\prime }S^{2}1$, trS, $trS^{2}$ and $y_{d}^{\prime }y_{d}$ in Equation (6) are expressed in terms of parameters $\sigma_{s}^{2}$, $\sigma_{r}^{2}$ and $\sigma_{e}^{2}$.

## APPENDIX E Power function in the case of $VP_{F}$

Starting from Equation (4), we have,

$$\begin{array}{cc} & P[L>\rho_{0}|\rho =\rho_{A}]\geq 1-\beta ,\\ & \;\Leftrightarrow \;P\left[\frac{1}{L}-1<\frac{1}{\rho_{0}}-1|\rho =\rho_{A}\right]\geq 1-\beta . \end{array}$$

Plugging in Equation (12) for the lower limit of the confidence interval in this case, we obtain,

$$P\left[\frac{{\hat{\sigma }}_{r}^{2}+{\hat{\sigma }}_{e}^{2}}{{\hat{\sigma }}_{s}^{2}F_{\hat{df_{1}},\hat{df_{2}}}(\alpha )}<\frac{1}{\rho_{0}}-1|\rho =\rho_{A}\right]\geq 1-\beta .$$

Since, after some algebraic manipulations starting from Equation (2), we obtain,

$${\left(\frac{1}{\hat{\rho }}-1\right)}^{-1}=\frac{{\hat{\sigma }}_{s}^{2}}{{\hat{\sigma }}_{r}^{2}+{\hat{\sigma }}_{e}^{2}},$$

we can rewrite the above expression on the left side as,

$$\begin{array}{c} P\left[\left(\frac{1}{\hat{\rho }}-1\right)\frac{1}{F_{\hat{df_{1}},\hat{df_{2}}}(\alpha )}<\frac{1}{\rho_{0}}-1|\rho =\rho_{A}\right]\geq 1-\beta , \end{array}$$

Note that under the alternative hypothesis, that is $\rho =\rho_{A}$, the degrees of freedom for the F− distribution are ${df}_{1}=\frac{2\sigma_{s}^{4}}{V({\hat{\sigma }}_{s}^{2})}$ and $df_{2}=\frac{2{\left(\sigma_{r}^{2}+\sigma_{e}^{2}\right)}^{2}}{V({\hat{\sigma }}_{r}^{2})+V({\hat{\sigma }}_{e}^{2})}$. The expressions for the variances of the variance estimators are obtained from Appendix B.

Since according to the $VP_{F}$ method, it is assumed that $\frac{{\hat{\sigma }}_{s}^{2}}{{\hat{\sigma }}_{r}^{2}+{\hat{\sigma }}_{e}^{2}}$ is distributed as ${\left(\frac{1}{\rho }-1\right)}^{-1}f_{df_{1},df_{2}}$, the power function can then be written as,

$$F_{{df}_{2},{df}_{1}}\left(\frac{\tau (\rho_{0})}{\tau (\rho_{A})}F_{{df}_{1},{df}_{2}}(\alpha )\right),$$

where $\tau (x)=\frac{1}{x}-1$.

## APPENDIX F Mean Squares between raters when considering random raters vs fixed raters

When the rater effects are considered random, the mean squares between raters ($MSR_{r}$) follow a distribution proportional to a $\chi_{k-1}^{2}$ distribution where k−1 is the degrees of freedom of the $\chi^{2}$ distribution (Mondal et al., 2025). Specifically,

$$MSR_{r}∼\chi_{k-1}^{2}\times \frac{n\sigma_{r}^{2}+\sigma_{e}^{2}}{k-1},$$

with $\sigma_{r}^{2}$, $\sigma_{e}^{2}$, n and k as defined in Equation (1).

When, instead, the rater effects are considered fixed, the mean squares between raters ($MSR_{f}$) follow a distribution proportional to a non‐central $\chi_{k-1}^{2}(\lambda )$ distribution where λ is the non‐centrality parameter (Bilodeau, 2022). Specifically,

$$MSR_{f}∼\chi_{k-1}^{2}(\lambda )\times \frac{\sigma_{e}^{2}}{k-1},$$

where $\lambda =\frac{n(k-1)\theta_{r}^{2}}{\sigma_{e}^{2}}$ with $\theta_{r}^{2}$ the average squared deviation of the rater effects analogous to the variance component $\sigma_{r}^{2}$ in the random‐effects model (McGraw & Wong, 1996).

The expected mean squares can be calculated as,

$$\begin{array}{ll} & E[MSR_{r}]=E\left[\chi_{k-1}^{2}\times \frac{n\sigma_{r}^{2}+\sigma_{e}^{2}}{k-1}\right]\;\\ & =(k-1)\times \frac{n\sigma_{r}^{2}+\sigma_{e}^{2}}{k-1}\;\\ & =n\sigma_{r}^{2}+\sigma_{e}^{2},\;\text{and}\;\\ & E[MSR_{f}]=E\left[\chi_{k-1}^{2}(\lambda )\times \frac{\sigma_{e}^{2}}{k-1}\right]\;\\ & =(k-1+\lambda )\times \frac{\sigma_{e}^{2}}{k-1}\;\\ & =\left(1+\frac{n\theta_{r}^{2}}{\sigma_{e}^{2}}\right)\sigma_{e}^{2}\;\\ & =n\theta_{r}^{2}+\sigma_{e}^{2}.\; \end{array}$$

The variances of the mean squares are calculated as,

$$\begin{array}{ll} & V[MSR_{r}]=V\left[\chi_{k-1}^{2}\times \frac{n\sigma_{r}^{2}+\sigma_{e}^{2}}{k-1}\right]\;\\ & =V[\chi_{k-1}^{2}]\times {\left(\frac{n\sigma_{r}^{2}+\sigma_{e}^{2}}{k-1}\right)}^{2}\;\\ & =2(k-1)\times {\left(\frac{n\sigma_{r}^{2}+\sigma_{e}^{2}}{k-1}\right)}^{2}\;\\ & =\frac{2{\left(n\sigma_{r}^{2}+\sigma_{e}^{2}\right)}^{2}}{k-1},\;\text{and}\; \end{array}$$

$$\begin{array}{ll} & V[MSR_{f}]=V\left[\chi_{k-1}^{2}(\lambda )\times \frac{\sigma_{e}^{2}}{k-1}\right]\;\\ & =V[\chi_{k-1}^{2}(\lambda )]\times {\left(\frac{\sigma_{e}^{2}}{k-1}\right)}^{2}\;\\ & =2(k-1+2\lambda )\times {\left(\frac{\sigma_{e}^{2}}{k-1}\right)}^{2}\;\\ & =2\left(k-1+\frac{2n(k-1)\theta_{r}^{2}}{\sigma_{e}^{2}}\right)\times {\left(\frac{\sigma_{e}^{2}}{k-1}\right)}^{2}\;\\ & =2\left(1+\frac{2n\theta_{r}^{2}}{\sigma_{e}^{2}}\right)\times \frac{{\left(\sigma_{e}^{2}\right)}^{2}}{k-1}\;\\ & =\frac{2(\sigma_{e}^{2}+2n\theta_{r}^{2})\times \sigma_{e}^{2}}{k-1}\;\\ & =\frac{2(\sigma_{e}^{4}+2n\theta_{r}^{2}\sigma_{e}^{2}+n^{2}\theta_{r}^{4})-2n^{2}\theta_{r}^{4}}{k-1}\;\\ & =\frac{2{\left(n\theta_{r}^{2}+\sigma_{e}^{2}\right)}^{2}}{k-1}-\frac{2n^{2}\theta_{r}^{4}}{k-1}.\; \end{array}$$

Therefore, although the point estimation of the ICC remains the same under both random‐ and fixed‐effects formulations, the variance of the mean squares between raters is smaller when raters are treated as fixed. As a result, the variance of the ICC estimator will also be smaller, leading to narrower confidence intervals. The lower limit of the confidence interval therefore moves closer to the point estimate when the rater effects are considered fixed rather than random. In the context of our hypothesis testing framework, this implies that the lower limit of the confidence interval is more likely to exceed the threshold $\rho_{0}$. Since the power is calculated as the probability that the lower limit of the confidence interval is greater than $\rho_{0}$, considering raters as fixed can therefore result in higher power for the test, given a number of participants and thereby lead to smaller required sample sizes to achieve the power 1−β.

However, it is important to note that the choice between treating rater effects as fixed or random reflects different considerations. Treating rater effects as fixed restricts the inference to the specific set of raters included in the study. In contrast, modelling rater effects as random supports generalization beyond the observed raters to a broader population of potential raters.
