# Supplementary material for: Sample size determination for hypothesis testing on the intraclass correlation coefficient in a two‐way analysis of variance model
Source: Br J Math Stat Psychol. 2025 Nov 14;79(3):590–620. doi: 10.1111/bmsp.70016 (PMC13679772; doi:10.1111/bmsp.70016)
Supplement: Supplementary file 1 [file BMSP-79-590-s001.zip › Supplementary file captions.docx]

1. File 1: Heatmaps summarizing the type-I error rates for ρ = 0.7 and ρ = 0.8 following Section 4.3 of the main manuscript.
2. File 2: Raw type-I error rates of the seven confidence interval methods across different values of n, k, R, and ρ mentioned in Section 4.3 of the main manuscript.
3. File 3: Results of the evaluation of the sample size determination procedures, including empirical type-I error rates and empirical power at the calculated sample sizes.
4. File 4: Values of P(n) for different combinations of n, k, R, ρ0, ρA, and α = 0.05, illustrating the monotonicity of the power function with respect to n.
