# Supplementary material for: Sample size determination for hypothesis testing on the intraclass correlation coefficient in a two‐way analysis of variance model
Source: Br J Math Stat Psychol. 2025 Nov 14;79(3):590–620. doi: 10.1111/bmsp.70016 (PMC13679772; doi:10.1111/bmsp.70016)

Supplementary file 1

A Results of the Simulation study for $\rho = 0.7$

Figure 1: Heatmap depicting the empirical type-I error rate from the Monte Carlo simulation study for a one-sided test on ρ at a 5% level, considering $\rho = 0.7$, $k = 2$ and $R \in \{0.01, 0.05, 0.1, 0.2, 0.3, 0.5, 0.6, 0.9, 1.0, 2.0, 5.0\}$. The distances from 0.05 are color-graded, with a '+' sign indicating that the empirical type-I error rate is greater than 0.05. A distance $[0, 0.01]$ indicates that the type-I error rate is within the interval $[0.04, 0.06]$; a distance $(0.01, 0.02]$ indicates that the type-I error rate is within $(0.06, 0.07]$ or $[0.03, 0.04)$; a distance $(0.02, 0.03]$ indicates that the type-I error rate is within $(0.07, 0.08]$ or $[0.02, 0.03)$; a distance $(0.03, 0.04]$ indicates that the type-I error rate is within $(0.08, 0.09]$ or $[0.01, 0.02)$ and a distance $(0.04, 0.05]$ indicates that the type-I error rate is within $(0.09, 0.10]$ or $[0, 0.01)$.

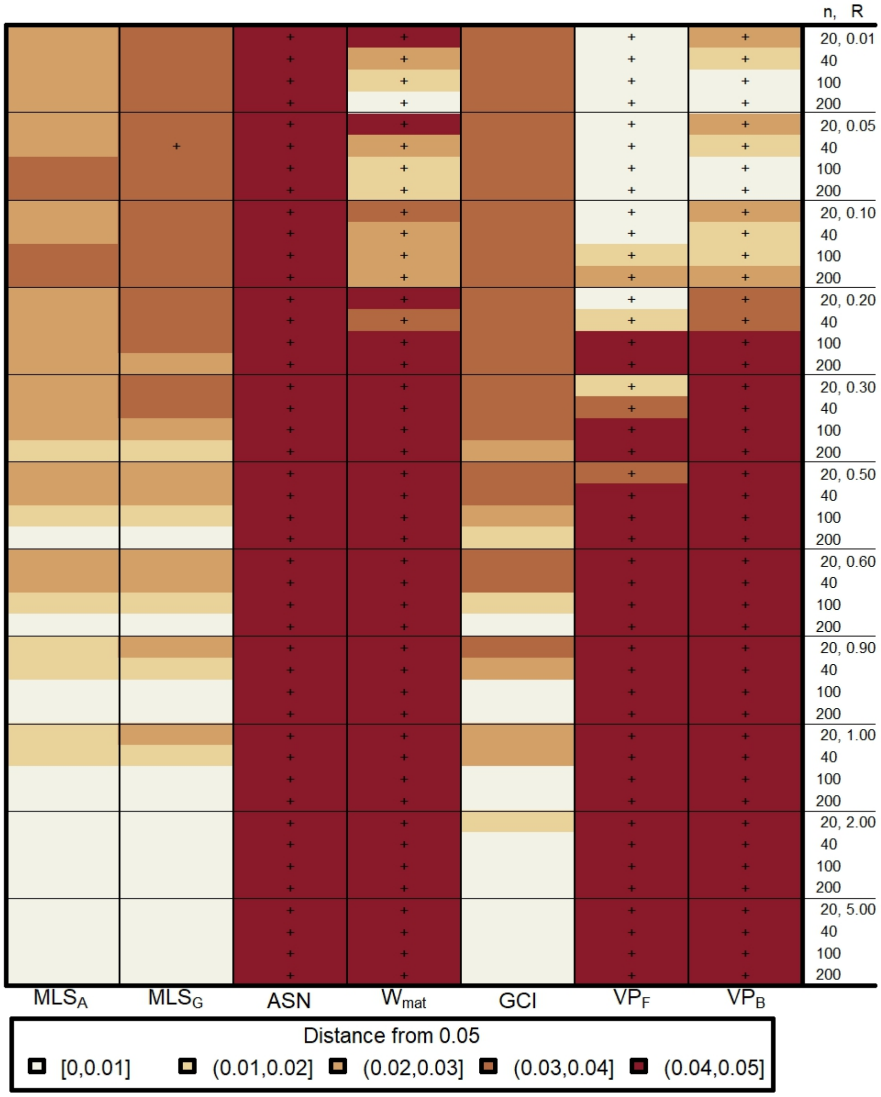

Figure 2: Heatmap depicting the empirical type-I error rate from the Monte Carlo simulation study for a one-sided test on ρ at a 5% level, considering $\rho = 0.7$, $k \in \{3, 4, 5, 8, 15\}$ and $R \in \{0.01, 0.05, 0.1, 0.2\}$. The distances from 0.05 are color-graded, with a '+' sign indicating that the empirical type-I error rate is greater than 0.05. A distance $[0, 0.01]$ indicates that the type-I error rate is within the interval $[0.04, 0.06]$; a distance $(0.01, 0.02]$ indicates that the type-I error rate is within $(0.06, 0.07]$ or $[0.03, 0.04)$; a distance $(0.02, 0.03]$ indicates that the type-I error rate is within $(0.07, 0.08]$ or $[0.02, 0.03)$; a distance $(0.03, 0.04]$ indicates that the type-I error rate is within $(0.08, 0.09]$ or $[0.01, 0.02)$ and a distance $(0.04, 0.05]$ indicates that the type-I error rate is within $(0.09, 0.10]$ or $[0, 0.01)$.

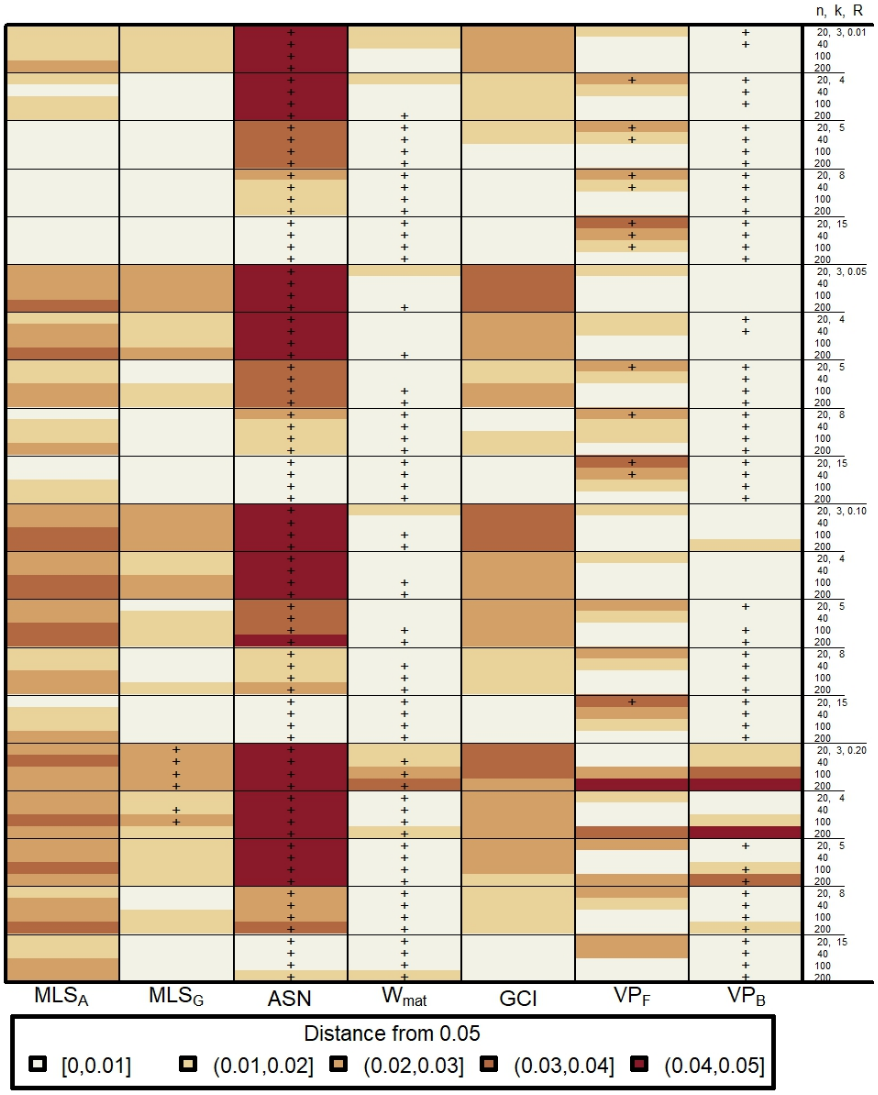

Figure 3: Heatmap depicting the empirical type-I error rate from the Monte Carlo simulation study for a one-sided test on ρ at a 5% level, considering $\rho = 0.7$, $k \in \{3, 4, 5, 8, 15\}$ and $R \in \{0.3, 0.5, 0.6, 0.9\}$. The distances from 0.05 are color-graded, with a '+' sign indicating that the empirical type-I error rate is greater than 0.05. A distance $[0, 0.01]$ indicates that the type-I error rate is within the interval $[0.04, 0.06]$; a distance $(0.01, 0.02]$ indicates that the type-I error rate is within $(0.06, 0.07]$ or $[0.03, 0.04)$; a distance $(0.02, 0.03]$ indicates that the type-I error rate is within $(0.07, 0.08]$ or $[0.02, 0.03)$; a distance $(0.03, 0.04]$ indicates that the type-I error rate is within $(0.08, 0.09]$ or $[0.01, 0.02)$ and a distance $(0.04, 0.05]$ indicates that the type-I error rate is within $(0.09, 0.10]$ or $[0, 0.01)$.

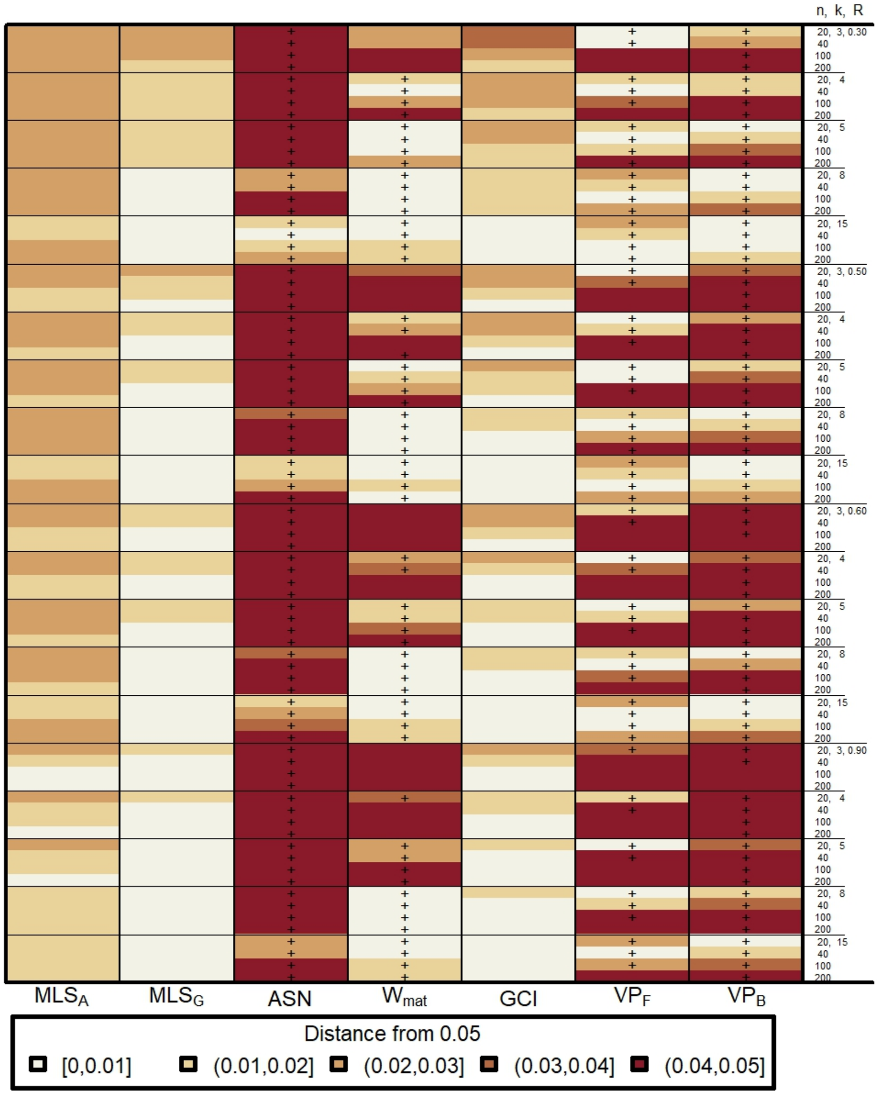

Figure 4: Heatmap depicting the empirical type-I error rate from the Monte Carlo simulation study for a one-sided test on ρ at a 5% level, considering $\rho = 0.7$, $k \in \{3, 4, 5, 8, 15\}$ and $R \in \{1, 2, 5\}$. The distances from 0.05 are color-graded, with a '+' sign indicating that the empirical type-I error rate is greater than 0.05. A distance $[0, 0.01]$ indicates that the type-I error rate is within the interval $[0.04, 0.06]$; a distance $(0.01, 0.02]$ indicates that the type-I error rate is within $(0.06, 0.07]$ or $[0.03, 0.04)$; a distance $(0.02, 0.03]$ indicates that the type-I error rate is within $(0.07, 0.08]$ or $[0.02, 0.03)$; a distance $(0.03, 0.04]$ indicates that the type-I error rate is within $(0.08, 0.09]$ or $[0.01, 0.02)$ and a distance $(0.04, 0.05]$ indicates that the type-I error rate is within $(0.09, 0.10]$ or $[0, 0.01)$.

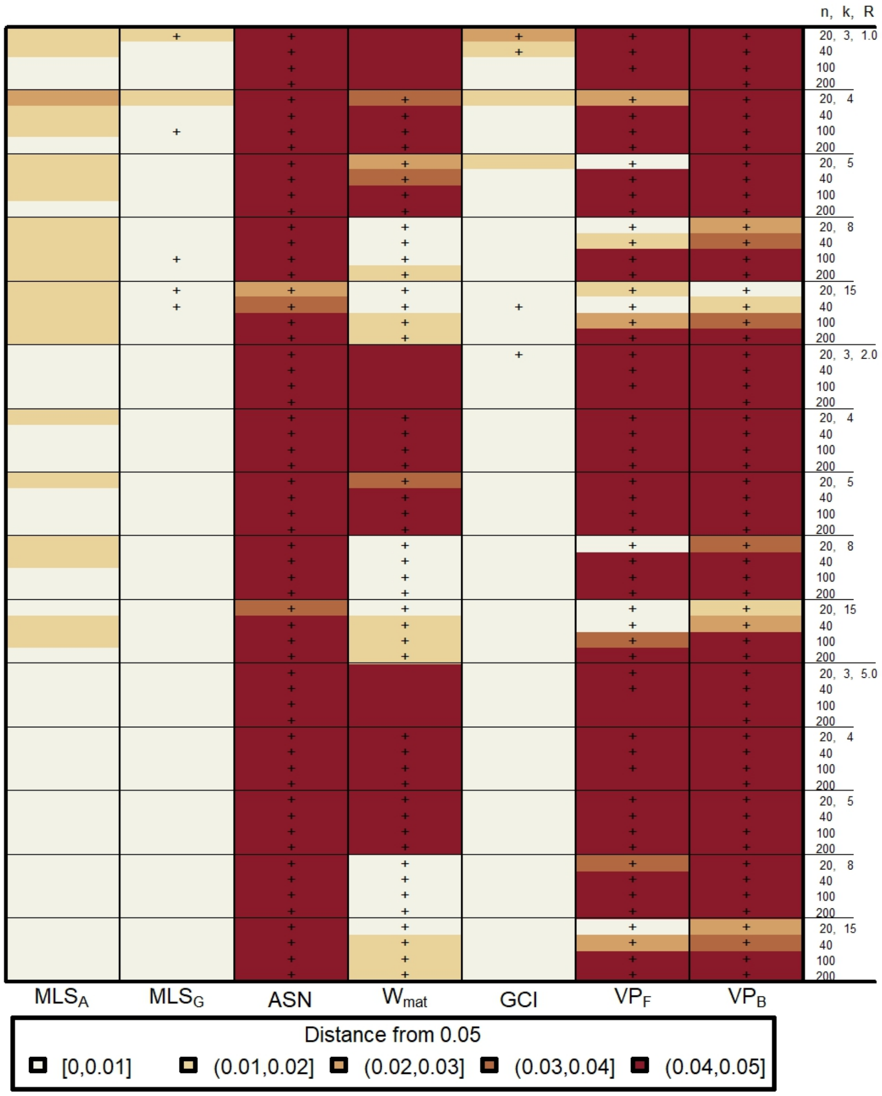

B Results of the Simulation study for $\rho = 0.8$

Figure 5: Heatmap depicting the empirical type-I error rate from the Monte Carlo simulation study for a one-sided test on ρ at a 5% level, considering $\rho = 0.8$, $k = 2$ and $R \in \{0.01, 0.05, 0.1, 0.2, 0.3, 0.5, 0.6, 0.9, 1.0, 2.0, 5.0\}$. The distances from 0.05 are color-graded, with a '+' sign indicating that the empirical type-I error rate is greater than 0.05. A distance $[0, 0.01]$ indicates that the type-I error rate is within the interval $[0.04, 0.06]$; a distance $(0.01, 0.02]$ indicates that the type-I error rate is within $(0.06, 0.07]$ or $[0.03, 0.04)$; a distance $(0.02, 0.03]$ indicates that the type-I error rate is within $(0.07, 0.08]$ or $[0.02, 0.03)$; a distance $(0.03, 0.04]$ indicates that the type-I error rate is within $(0.08, 0.09]$ or $[0.01, 0.02)$ and a distance $(0.04, 0.05]$ indicates that the type-I error rate is within $(0.09, 0.10]$ or $[0, 0.01)$.

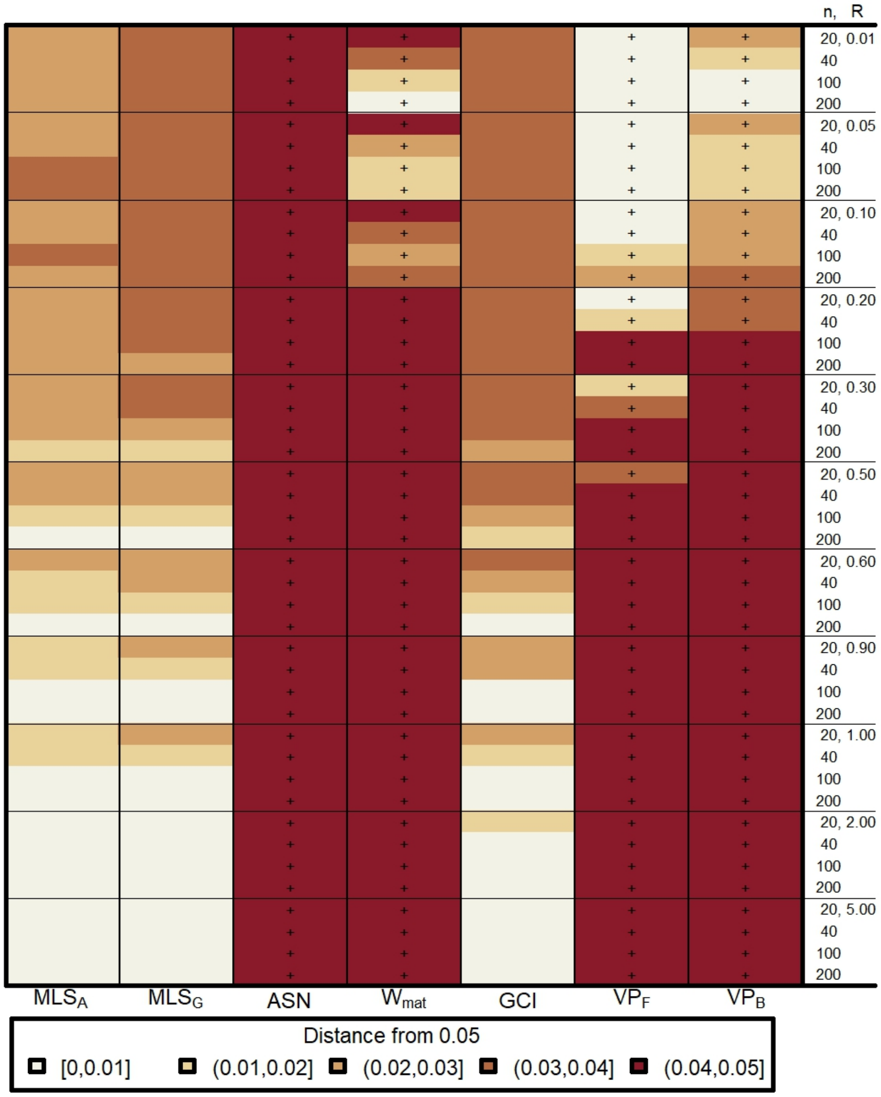

Figure 6: Heatmap depicting the empirical type-I error rate from the Monte Carlo simulation study for a one-sided test on ρ at a 5% level, considering $\rho = 0.8$, $k \in \{3, 4, 5, 8, 15\}$ and $R \in \{0.01, 0.05, 0.1, 0.2\}$. The distances from 0.05 are color-graded, with a '+' sign indicating that the empirical type-I error rate is greater than 0.05. A distance $[0, 0.01]$ indicates that the type-I error rate is within the interval $[0.04, 0.06]$; a distance $(0.01, 0.02]$ indicates that the type-I error rate is within $(0.06, 0.07]$ or $[0.03, 0.04)$; a distance $(0.02, 0.03]$ indicates that the type-I error rate is within $(0.07, 0.08]$ or $[0.02, 0.03)$; a distance $(0.03, 0.04]$ indicates that the type-I error rate is within $(0.08, 0.09]$ or $[0.01, 0.02)$ and a distance $(0.04, 0.05]$ indicates that the type-I error rate is within $(0.09, 0.10]$ or $[0, 0.01)$.

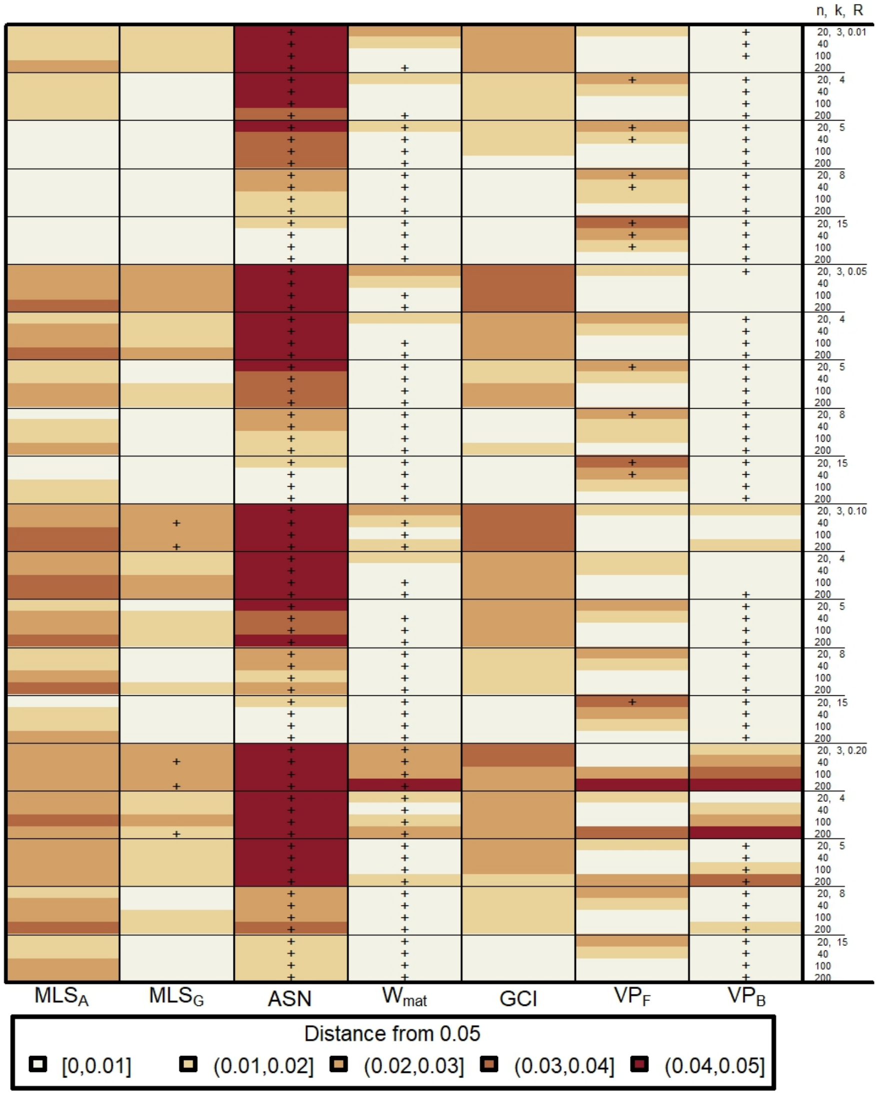

Figure 7: Heatmap depicting the empirical type-I error rate from the Monte Carlo simulation study for a one-sided test on ρ at a 5% level, considering $\rho = 0.8$, $k \in \{3, 4, 5, 8, 15\}$ and $R \in \{0.3, 0.5, 0.6, 0.9\}$. The distances from 0.05 are color-graded, with a '+' sign indicating that the empirical type-I error rate is greater than 0.05.A distance $[0, 0.01]$ indicates that the type-I error rate is within the interval $[0.04, 0.06]$; a distance $(0.01, 0.02]$ indicates that the type-I error rate is within $(0.06, 0.07]$ or $[0.03, 0.04)$; a distance $(0.02, 0.03]$ indicates that the type-I error rate is within $(0.07, 0.08]$ or $[0.02, 0.03)$; a distance $(0.03, 0.04]$ indicates that the type-I error rate is within $(0.08, 0.09]$ or $[0.01, 0.02)$ and a distance $(0.04, 0.05]$ indicates that the type-I error rate is within $(0.09, 0.10]$ or $[0, 0.01)$.

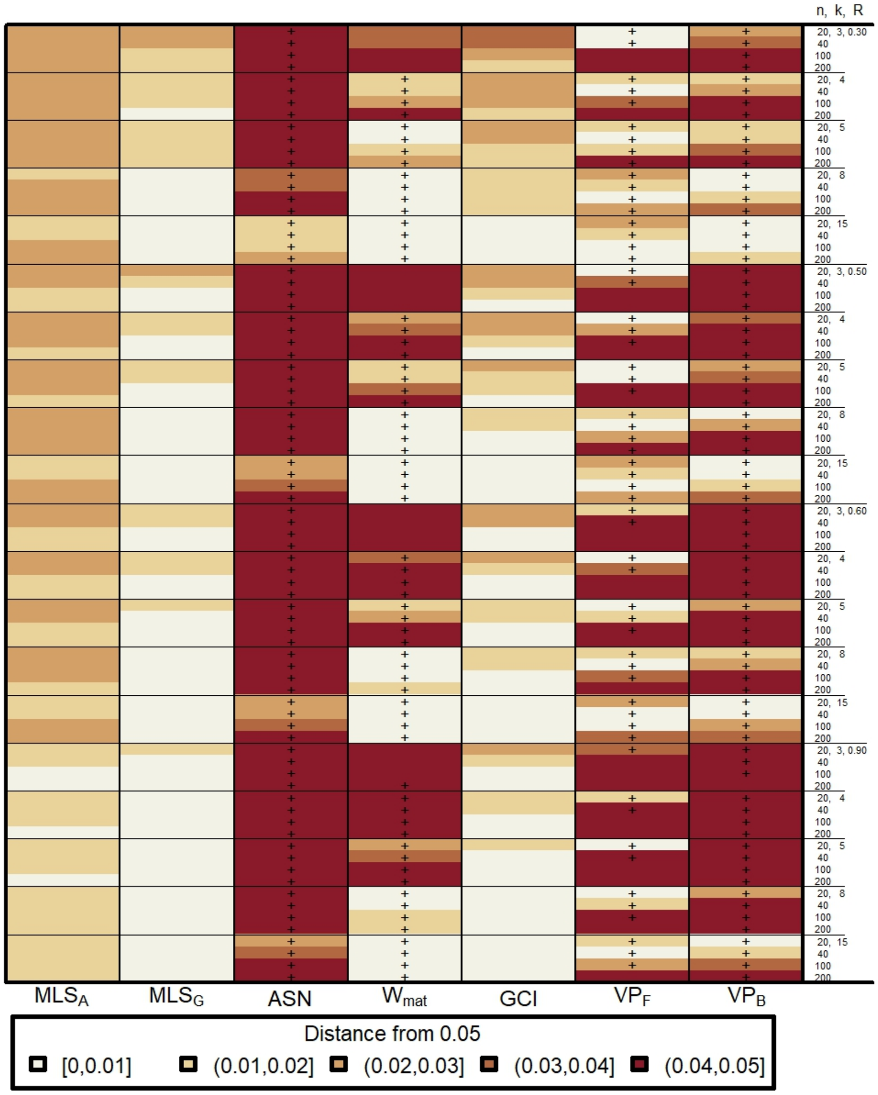

Figure 8: Heatmap depicting the empirical type-I error rate from the Monte Carlo simulation study for a one-sided test on ρ at a 5% level, considering $\rho = 0.8$, $k \in \{3, 4, 5, 8, 15\}$ and $R \in \{1, 2, 5\}$. The distances from 0.05 are color-graded, with a '+' sign indicating that the empirical type-I error rate is greater than 0.05. A distance $[0, 0.01]$ indicates that the type-I error rate is within the interval $[0.04, 0.06]$; a distance $(0.01, 0.02]$ indicates that the type-I error rate is within $(0.06, 0.07]$ or $[0.03, 0.04)$; a distance $(0.02, 0.03]$ indicates that the type-I error rate is within $(0.07, 0.08]$ or $[0.02, 0.03)$; a distance $(0.03, 0.04]$ indicates that the type-I error rate is within $(0.08, 0.09]$ or $[0.01, 0.02)$ and a distance $(0.04, 0.05]$ indicates that the type-I error rate is within $(0.09, 0.10]$ or $[0, 0.01)$.

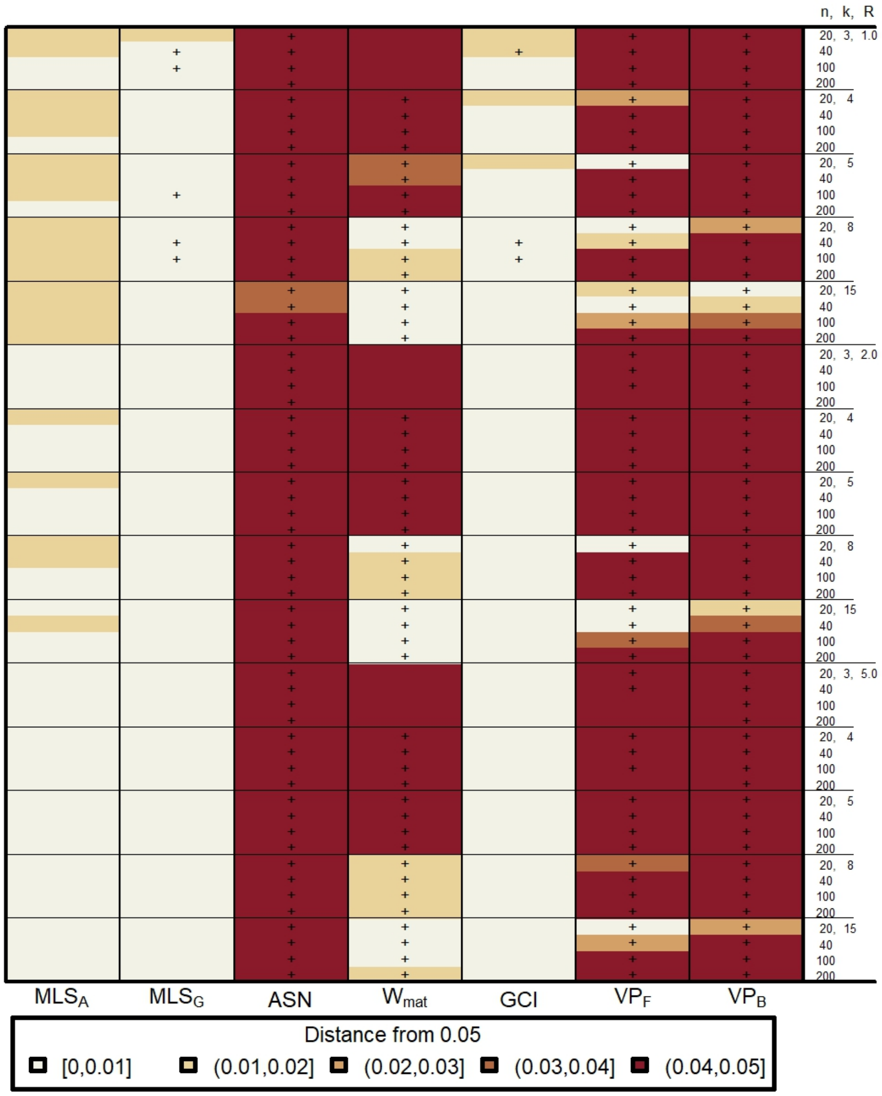

Supplement: Supplementary file 1 [file BMSP-79-590-s001.zip › supplementary_file_1.pdf]
